# Supplementary material for: Myogenic differentiation-derived small extracellular vesicles as a therapeutic strategy for miR-193b-3p-mediated muscle regeneration
Source: Mol Ther Nucleic Acids. 2026 Sep 3;37(4):103087. doi: 10.1016/j.omtn.2026.103087 (PMC13626940; doi:10.1016/j.omtn.2026.103087)
Supplement: Supplementary file 1 [file mmc1.pdf]

OMTN, Volume *37*

Supplemental information

Myogenic differentiation-derived small extracellular vesicles as a therapeutic strategy for miR-193b-3p-mediated muscle regeneration

Sujin Choi, Min-Ji Sung, Hyun-Ju An, and Soonchul Lee

Table S1. Primers used in this study (provided as a separate Excel file).

Table S2. List of DEMs in sEV-ED compared with sEV-ND (provided as a separate Excel file).

Table S3. List of DEMs in sEV-LD compared with sEV-ND (provided as a separate Excel file).

Table S4. The common list of DEMs between sEV-ED vs. sEV-ND and sEV-LD vs. sEV-ND.

Gene symbol	Fold change		p-value	
	sEV-ED/ sEV-ND	sEV-LD/ sEV-ND	sEV-ED/ sEV-ND	sEV-LD/ sEV-ND
hsa-miR-193b-3p	26.7408	45.5644	0.0005	0.0019
hsa-miR-23a-3p	4.7919	17.6417	0.0021	0.0047
hsa-miR-370-5p	9.9262	17.5993	0.0040	0.0032
hsa-miR-6849-5p	9.9387	13.2359	0.0090	0.0038
hsa-miR-206	10.2581	12.2363	0.0050	0.0083
hsa-miR-376b-3p	6.9722	9.7388	0.0030	0.0007
hsa-miR-484	3.2981	6.6408	0.0034	0.0082
hsa-miR-224-5p	4.6629	6.5526	0.0051	0.0013
hsa-miR-4301	4.2666	6.4162	0.0053	0.0020
hsa-miR-1296-5p	11.9674	5.9637	0.0028	0.0003
hsa-miR-323b-3p	3.6983	5.8763	0.0052	0.0068
hsa-miR-656-3p	3.2233	4.7527	0.0009	0.0001
hsa-miR-323a-3p	3.3752	3.9308	0.0023	0.0059
hsa-miR-941	3.9232	3.1788	0.0044	0.0008
hsa-miR-3074-5p	3.2300	2.8548	0.0020	0.0031
hsa-miR-139-5p	0.2696	0.3427	0.0088	0.0045

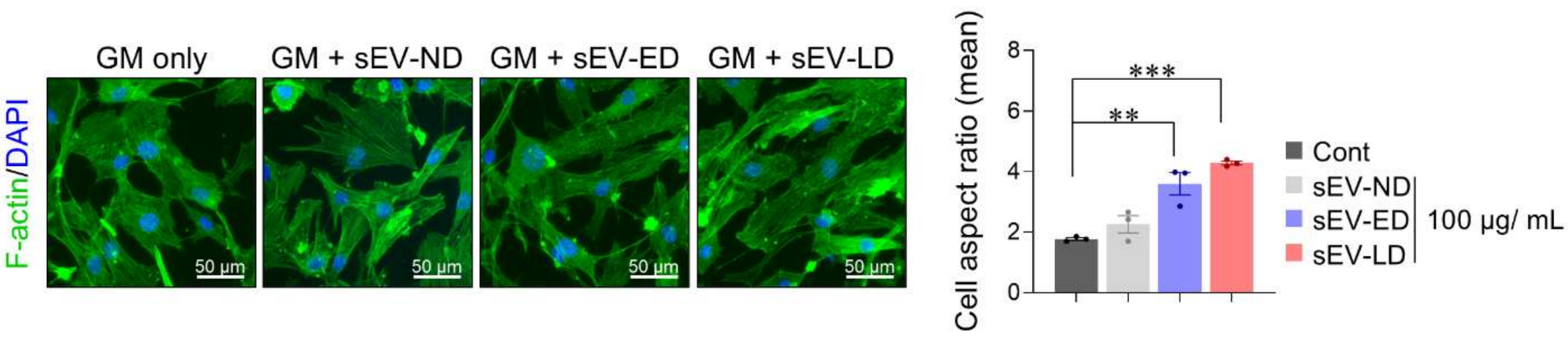

Figure S1. Immunofluorescence images of HSkMCs stained with F-actin (green) and DAPI (blue), and quantification of cell aspect ratio under growth medium conditions with sEV treatment.

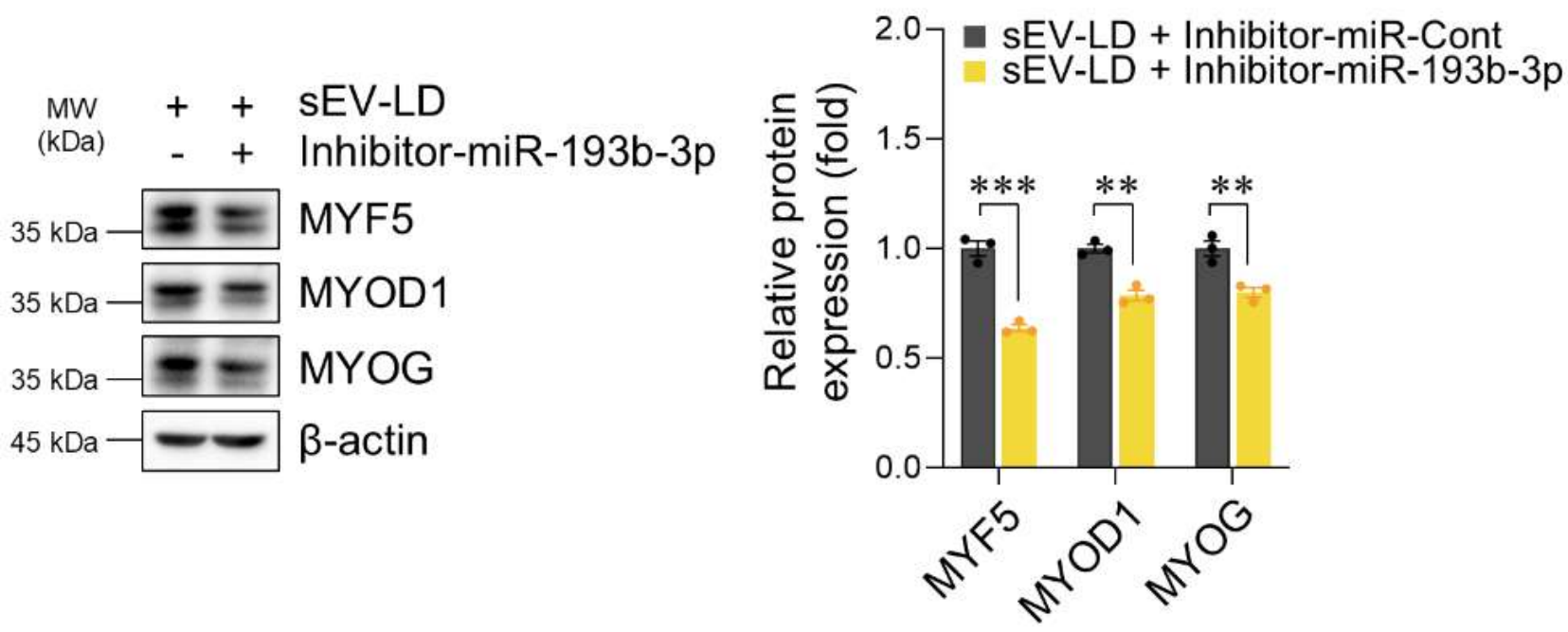

Figure S2. Western blot analysis of MYF5, MYOD1, and MYOG in HSkMCs co-treated with sEV-LD and either inhibitor-miR-Cont or inhibitor-miR-193b-3p.

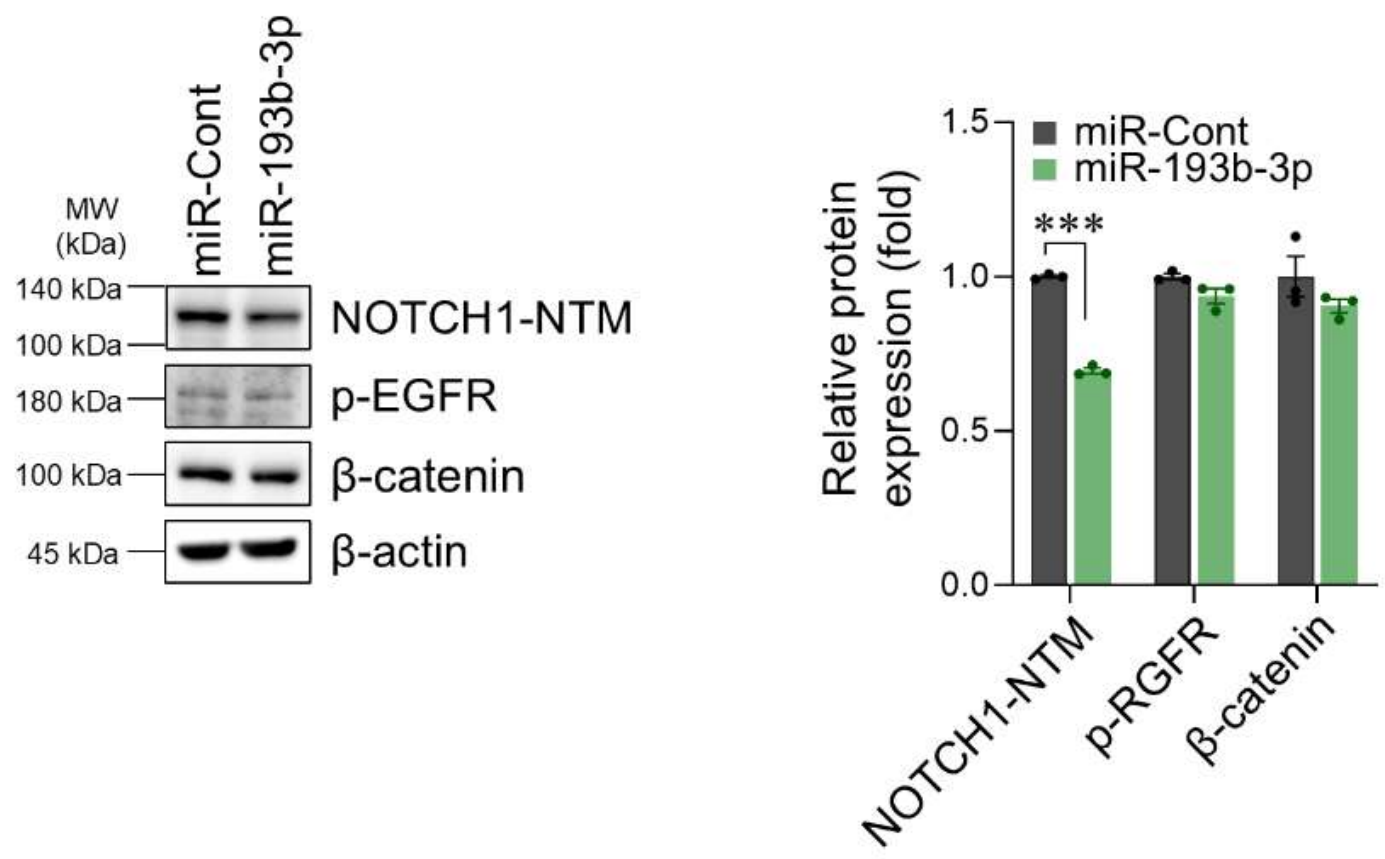

Figure S3. Western blot analysis of NOTCH1-NTM, p-EGFR, and β-catenin in HSkMCs transfected with either miR-Cont or miR-193b-3p.

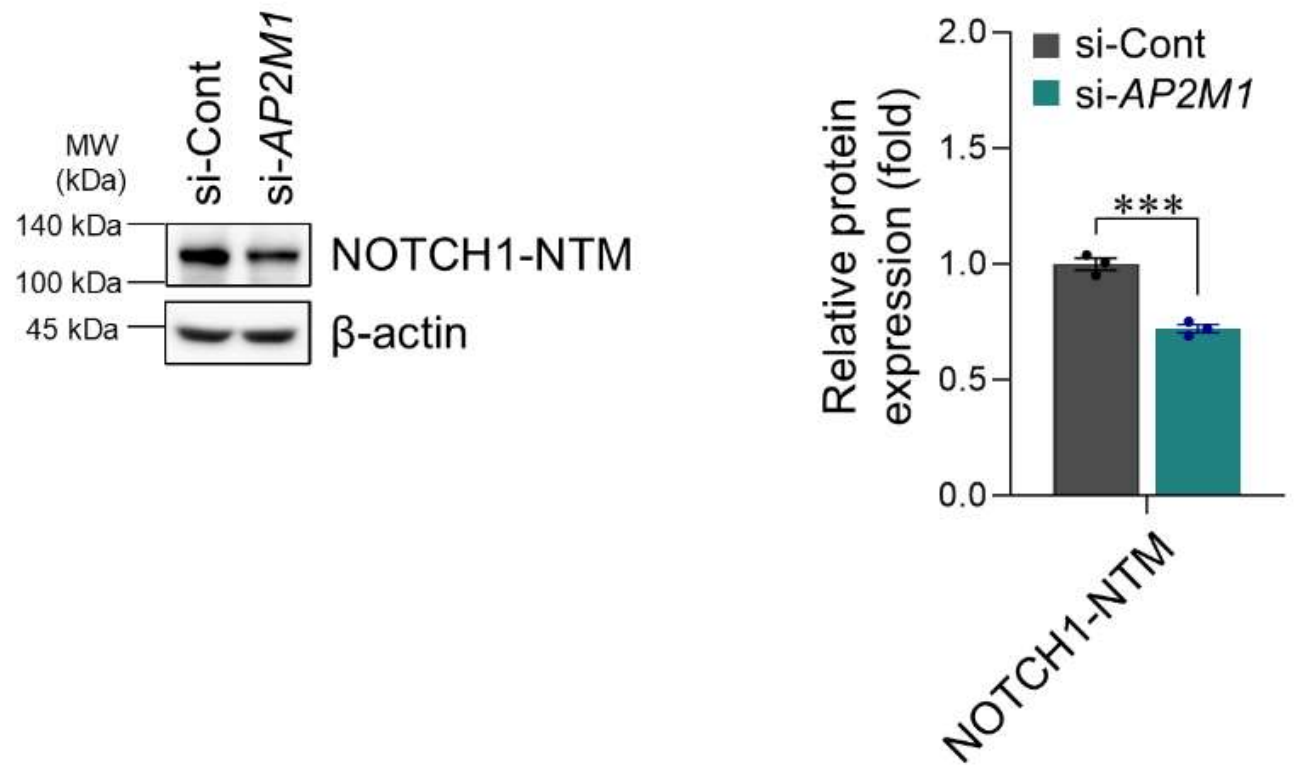

Figure S4. Western blot analysis of NOTCH1-NTM in HSkMCs transfected with either si-Cont or si-*AP2M1*.

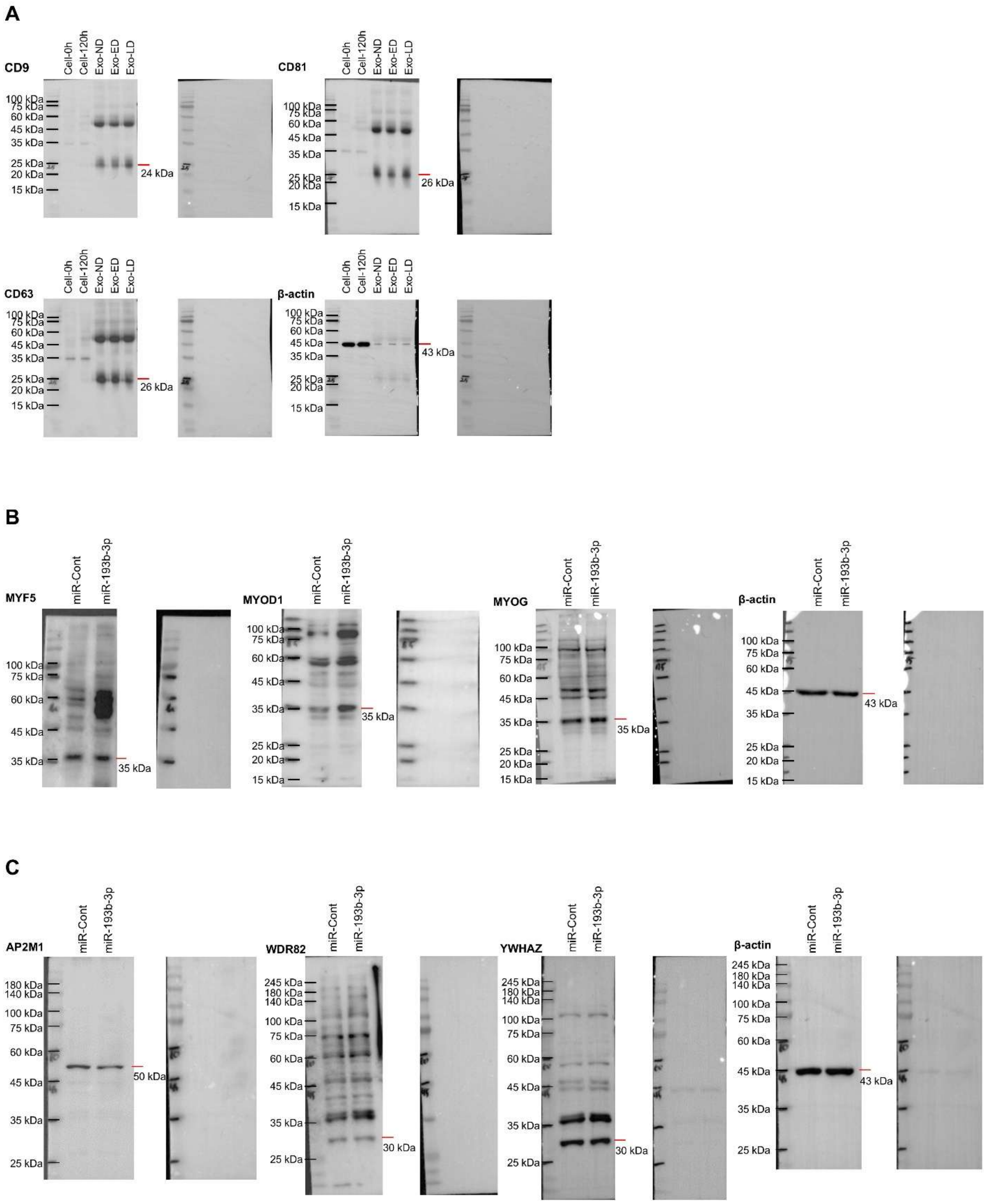
A
CD9
CD81
CD63
β-actin
Cell-0h
Cell-120h
Exo-ND
Exo-ED
Exo-LD
24 kDa
26 kDa
26 kDa
43 kDa
B
MYF5
MYOD1
MYOG
β-actin
miR-Cont
miR-193b-3p
35 kDa
35 kDa
35 kDa
43 kDa
C
AP2M1
WDR82
YWHAZ
β-actin
miR-Cont
miR-193b-3p
50 kDa
30 kDa
30 kDa
43 kDa

D

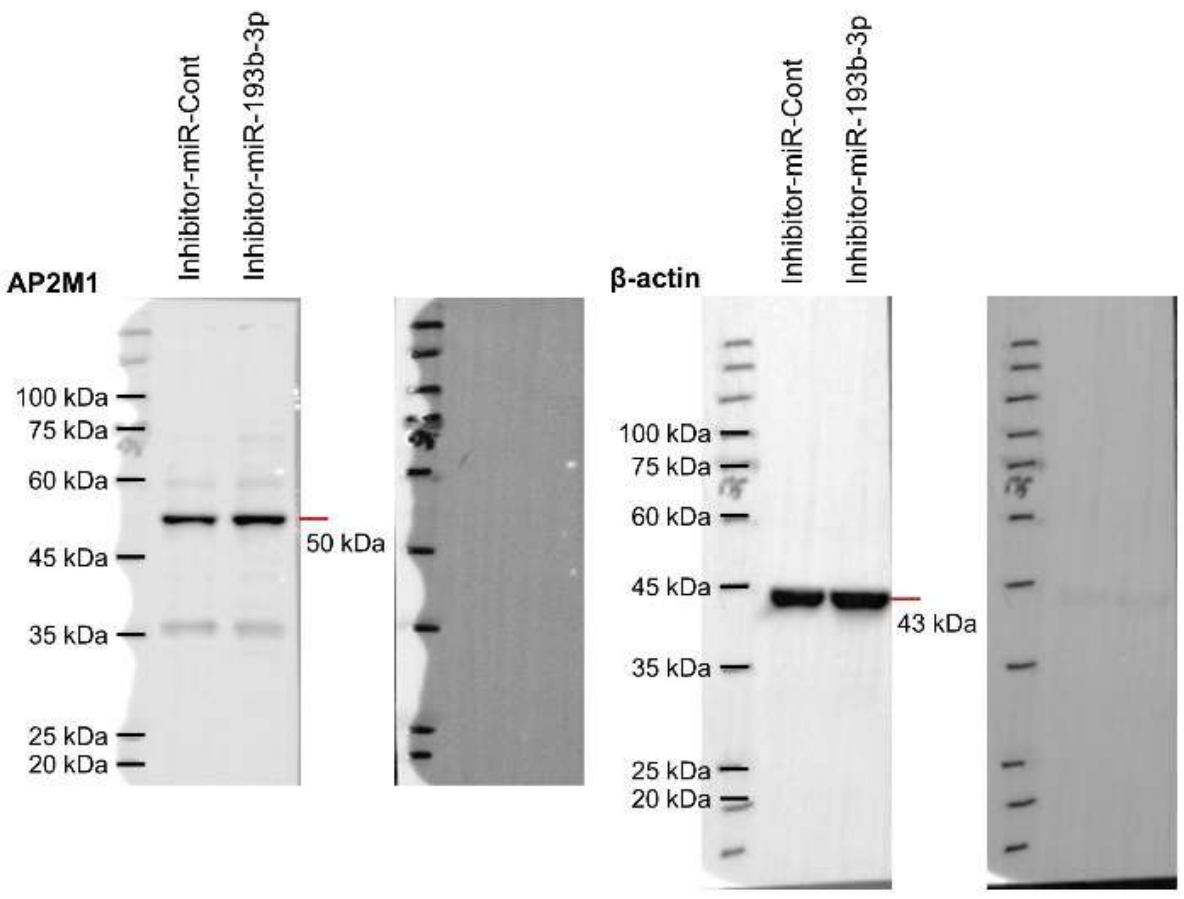
Inhibitor-miR-Cont
Inhibitor-miR-193b-3p
AP2M1
100 kDa
75 kDa
60 kDa
45 kDa
35 kDa
25 kDa
20 kDa
50 kDa
β-actin
Inhibitor-miR-Cont
Inhibitor-miR-193b-3p
100 kDa
75 kDa
60 kDa
45 kDa
35 kDa
25 kDa
20 kDa
43 kDa

E

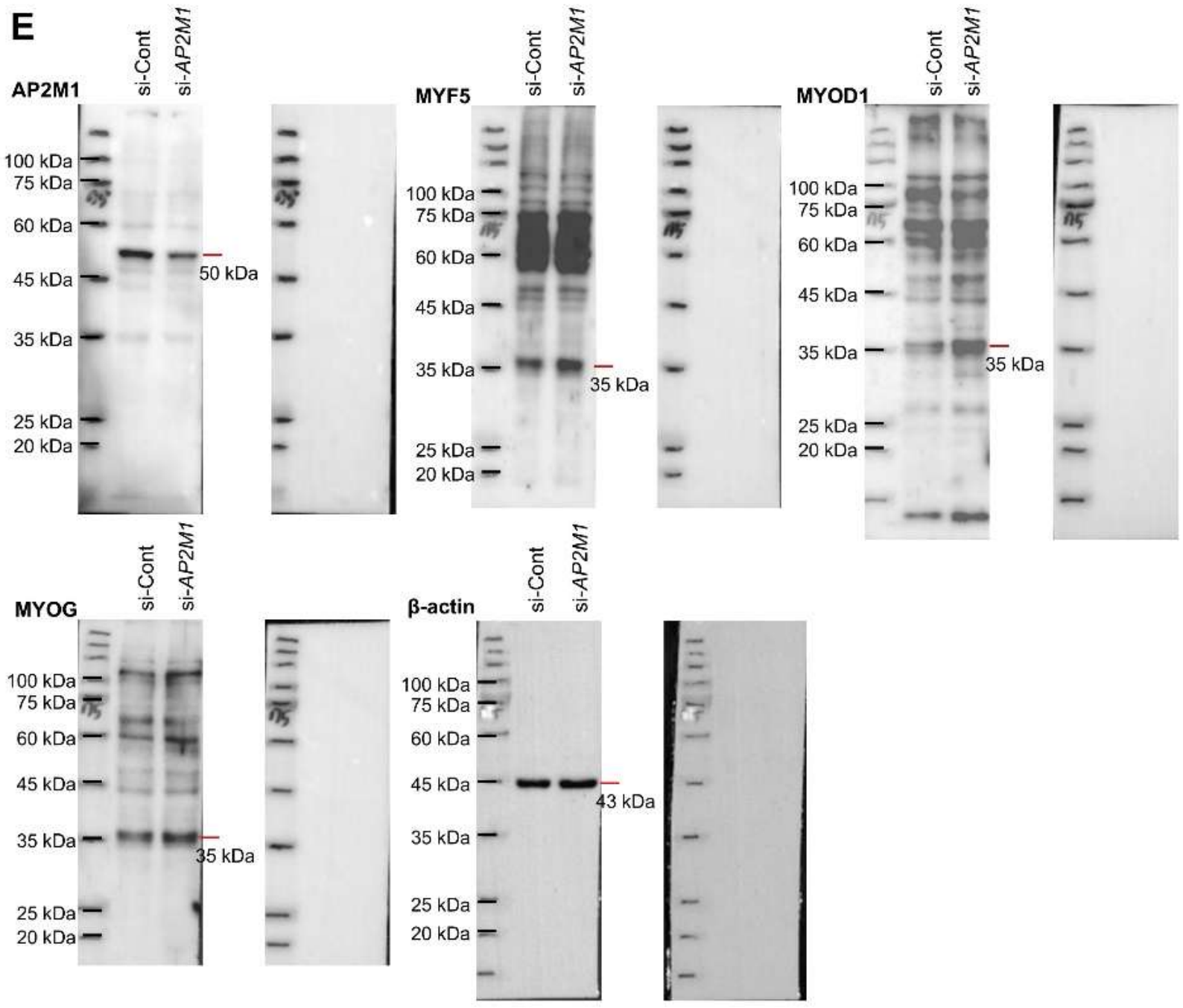
AP2M1
si-Cont
si-*AP2M1*
100 kDa
75 kDa
60 kDa
45 kDa
35 kDa
25 kDa
20 kDa
50 kDa
MYF5
si-Cont
si-*AP2M1*
100 kDa
75 kDa
60 kDa
45 kDa
35 kDa
25 kDa
20 kDa
35 kDa
MYOD1
si-Cont
si-*AP2M1*
100 kDa
75 kDa
60 kDa
45 kDa
35 kDa
25 kDa
20 kDa
35 kDa
MYOG
si-Cont
si-*AP2M1*
100 kDa
75 kDa
60 kDa
45 kDa
35 kDa
25 kDa
20 kDa
35 kDa
β-actin
si-Cont
si-*AP2M1*
100 kDa
75 kDa
60 kDa
45 kDa
35 kDa
25 kDa
20 kDa
43 kDa

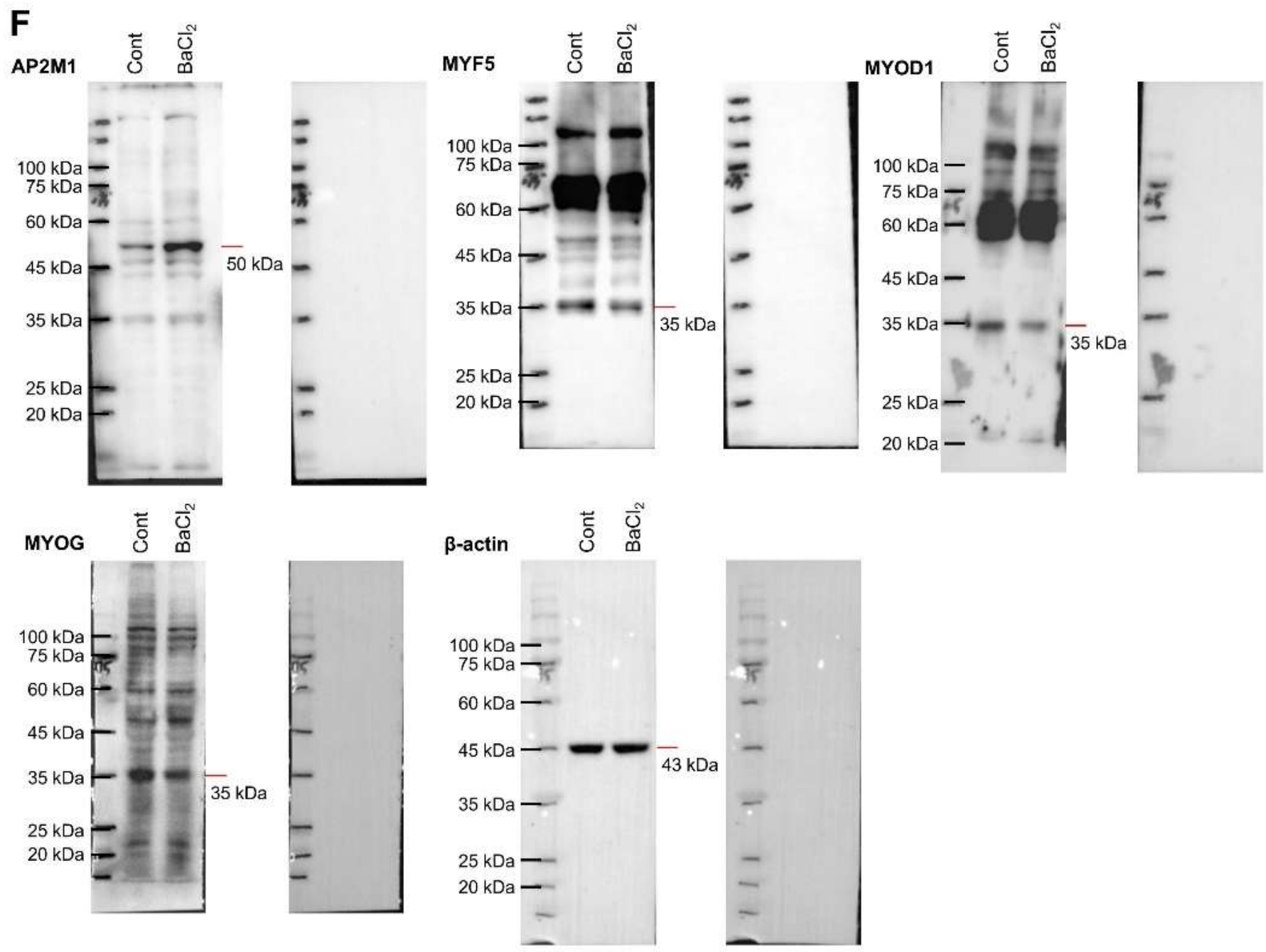

Figure S5. Uncropped western blot images correspond to the main figures.
