# Supplementary material for: Myogenic differentiation-derived small extracellular vesicles as a therapeutic strategy for miR-193b-3p-mediated muscle regeneration
Source: Mol Ther Nucleic Acids. 2026 Sep 3;37(4):103087. doi: 10.1016/j.omtn.2026.103087 (PMC13626940; doi:10.1016/j.omtn.2026.103087)
Supplement: Supplementary file 2 [file mmc5.pdf]

Molecular Therapy
Nucleic Acids
Original Article

Myogenic differentiation-derived small extracellular vesicles as a therapeutic strategy for miR-193b-3p-mediated muscle regeneration

Sujin Choi,[1] Min-Ji Sung,[2] Hyun-Ju An,[1] and Soonchul Lee[1]

[1]Department of Orthopedic Surgery, CHA University School of Medicine, Gyeonggi-do, South Korea; [2]Division of Nephrology, CHA University School of Medicine, Gyeonggi-do, South Korea

The regeneration of skeletal muscle involves precisely regulated intercellular communication, including extracellular vesicle (EV)-mediated processes. In this study, we show that during *in vitro* myogenic differentiation of human skeletal muscle progenitor cells (myoblasts), miR-193b-3p accumulates within small EVs (sEVs) in a temporally regulated manner. Both sEV-mediated and synthetic delivery of miR-193b-3p enhanced myogenic differentiation *in vitro* and promoted histological recovery in a murine volumetric muscle loss model. Mechanistically, we demonstrate that miR-193b-3p directly suppresses adapter-related protein complex 2 subunit mu 1 (AP2M1), a clathrin-associated adapter protein that has not been well characterized in muscle biology, and identify it as a negative regulator of myogenic progression. AP2M1 knockdown phenocopied the effects of miR-193b-3p, suggesting that this regulatory axis may contribute to muscle regeneration. In addition, transcriptomic analyses of aged rodent and sarcopenic human datasets revealed conserved upregulation of AP2M1 in degenerative muscle states, supporting its potential clinical relevance. Collectively, these findings reveal a myogenic differentiation-dependent, sEV-mediated miRNA regulatory mechanism that contributes to muscle regeneration. Based on these results, we propose the miR-193b-3p-AP2M1 axis as a potential therapeutic target for muscle-wasting disorders.

INTRODUCTION

Skeletal muscles are characterized by a strong capacity for regeneration, primarily mediated by quiescent satellite cells that are rapidly activated in response to injury to initiate a tightly orchestrated cascade of proliferation, lineage commitment, and terminal myogenic differentiation.[1–3] Although this endogenous regenerative program efficiently restores muscle structure following minor injuries, it is often insufficient under severe pathological conditions such as volumetric muscle loss (VML), chronic trauma, and age-associated degenerative diseases such as sarcopenia.[4–6] These limitations highlight the need to identify molecular drivers that can enhance or restore myogenic potential, ultimately contributing to the development of therapeutic strategies.

MicroRNAs (miRNAs) have emerged as important regulators of skeletal muscle biology, functioning as fine-tuners of gene expression programs that dictate the fate of satellite cells, myotube formation, and regenerative capacity.[7] Classical muscle-enriched miRNAs, such as miR-1, miR-133, and miR-206, have been established to modulate key signaling pathways involved in lineage specification and fusion during myogenesis.[8–10] For example, miR-206 promotes satellite cell differentiation and accelerates muscle regeneration following acute injury,[11–13] whereas miR-1 and miR-133 play key roles in balancing proliferation and differentiation.[8,13,14] However, although these studies highlight the therapeutic potential of miRNA-based modulation of muscle repair, the repertoire of miRNAs involved in coordinating muscle regeneration, particularly those acting as paracrine factors, remains incompletely understood.

Among the emerging mediators of intercellular communication, extracellular vesicles (EVs), particularly small EVS (sEVs), have gained increasing attention.[15,16] These vesicles are nano-sized particles released by donor cells that serve as carriers of bioactive molecules, including miRNAs, proteins, and lipids, enabling them to influence the behavior of recipient cells.[16,17] Recent studies have shown that sEVs derived from mesenchymal stem cells or endothelial progenitor cells can enhance angiogenesis, attenuate fibrosis, and promote muscle regeneration, supporting their reparative potential.[18,19] However, despite these advances, the dynamic sEV-mediated signaling that occurs during the myogenic differentiation of myoblasts, which models aspects of the physiological process of muscle repair, remains poorly understood.

Stage-specific sEV-associated miRNAs, whose expression is dynamically regulated during myogenic differentiation, are of particular interest given their potential roles as paracrine mediators of muscle

Received 22 December 2025; accepted 2 September 2026;
https://doi.org/10.1016/j.omtn.2026.103087.

Correspondence: Hyun-Ju An, Department of Orthopedic Surgery, CHA University School of Medicine, Gyeonggi-do, South Korea.
E-mail: yks486ahj@naver.com
Correspondence: Soonchul Lee, Department of Orthopedic Surgery, CHA University School of Medicine, Gyeonggi-do, South Korea.
E-mail: lsceline@cha.ac.kr

© 2026 The Authors.
Published by Elsevier Inc. on behalf of The American Society of Gene and Cell Therapy.
This is an open access article under the CC BY license (http://creativecommons.org/licenses/by/4.0/).

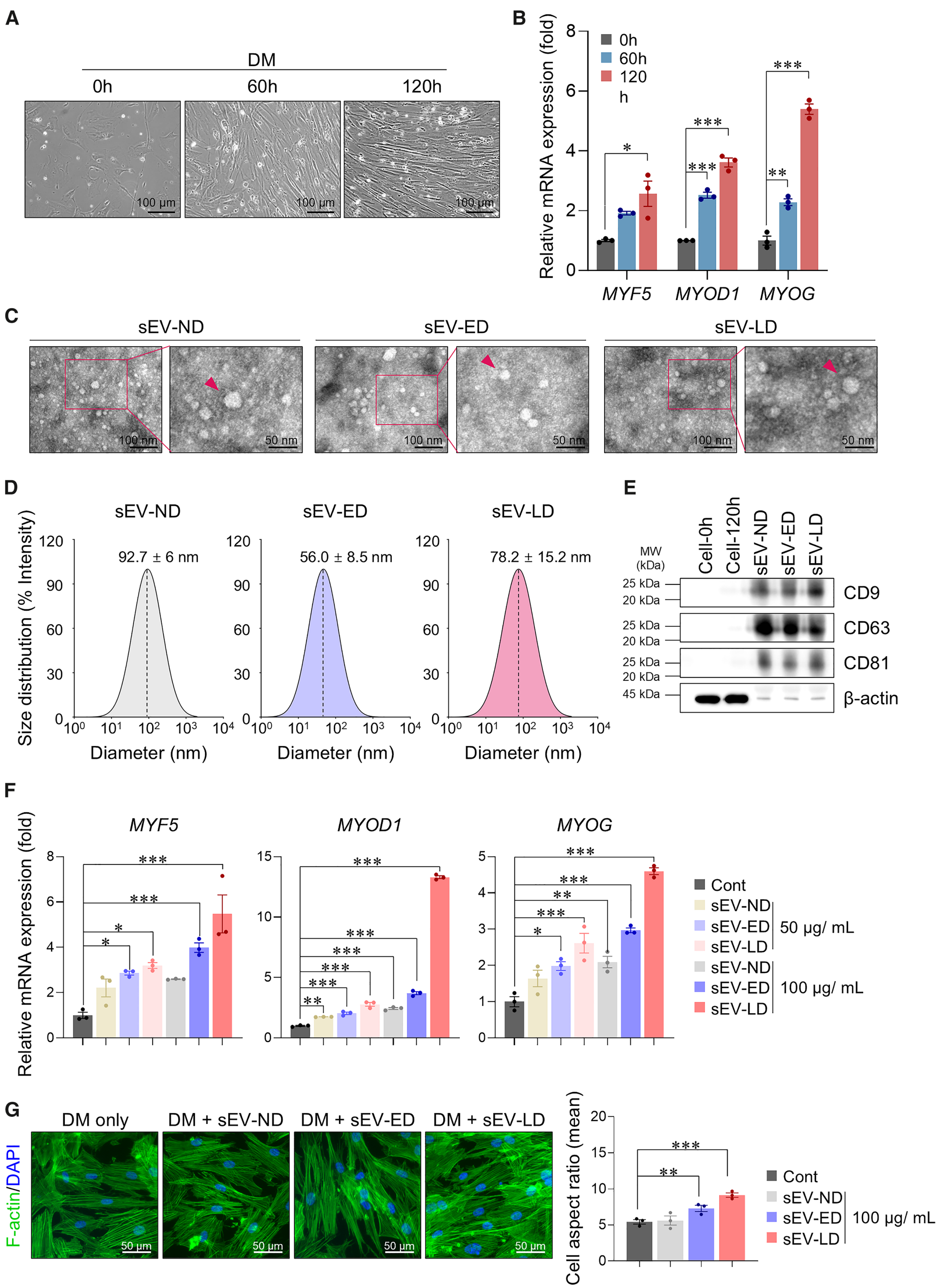

(legend on next page)

regeneration.[20,21] Systematic identification of these miRNAs may reveal previously uncharacterized molecular mechanisms governing muscle repair and provide novel therapeutic targets for muscle-wasting conditions. In this study, we profiled sEV-associated miRNA cargo released during myogenic differentiation and assessed their functional relevance in skeletal muscle regeneration. We identified a previously uncharacterized sEV-associated miRNA as a regulator of myogenesis. These findings enhance our understanding of sEV-mediated muscle regeneration and provide a basis for the development of miRNA-based therapeutic strategies.

RESULTS

Characterization of stage-specific sEVs and their pro-myogenic effects on HSkMCs

To obtain stage-specific sEVs released during myogenic differentiation, we used primary human skeletal muscle cells (HSkMCs). To establish a cellular model of myogenic differentiation, cells were cultured in differentiation medium (DM) for up to 120 h, resulting in progressive morphological changes indicative of myoblast fusion and myotube formation (Figure 1A). Myogenic differentiation was confirmed by a time-dependent increase in the expression of myogenic markers myogenic factor 5 (*MYF5*), myogenic differentiation 1 (*MYOD1*), and myogenin (*MYOG*), as assessed by quantitative reverse-transcription polymerase chain reaction (RT-qPCR) (Figure 1B).

sEVs were isolated from conditioned media collected no differentiation (sEV-ND, 60 h in growth medium), early differentiation (sEV-ED, 0–60 h in DM), and late differentiation (sEV-LD, 60–120 h in DM) conditions. In all vesicle groups, transmission electron microscopy (TEM) revealed vesicles with a typical exosome-like morphology (Figure 1C). Dynamic light scattering (DLS) analysis revealed distinct differences in particle size distributions among vesicle types, with modal diameters of 92.7 ± 6, 56.0 ± 8.5, and 78.2 ± 15.2 nm for sEV-ND, sEV-ED, and sEV-LD, respectively (Figure 1D). Western blot analysis confirmed that all vesicle preparations were positive for the canonical sEV markers CD9, CD63, and CD81 (Figure 1E). Overall, vesicles collected at each time point met the established criteria for sEVs based on TEM, DLS, and marker analyses.

To assess the pro-myogenic effects of different sEV fractions, naive myoblasts were exposed to each vesicle type at two concentrations (50 and 100 μg/mL) during early differentiation. All three sEV types induced dose-dependent increases in *MYF5*, *MYOD1*, and *MYOG* expression. Notably, at both concentrations, sEV-LD elicited the highest expression levels, indicating stronger pro-myogenic effects compared with earlier-stage sEVs (Figure 1F). To further evaluate the morphological consequences of differentiation, F-actin immunofluorescence staining was performed following treatment with 100 μg/mL sEVs. Under differentiation conditions, treatment with sEV-ED and sEV-LD promoted elongation-like morphological changes compared with DM alone, with the most pronounced effects observed in the sEV-LD group. Quantitative analysis confirmed a significant increase in cell aspect ratio following sEV-ED and sEV-LD treatment (Figure 1G). To compare the differentiation-inducing effects of sEVs with those of differentiation medium alone, HSkMCs were treated with sEVs under growth conditions. Although sEV treatment induced elongation-like morphological changes, these were less pronounced than those observed under differentiation conditions (Figure S1), suggesting that sEV treatment alone is insufficient to induce complete myogenic differentiation. Overall, these results demonstrate that sEVs released at different stages of differentiation vary in their capacity to promote pro-myogenic responses.

sEVs secreted during myogenic differentiation enhance skeletal muscle regeneration following volumetric injury

To evaluate the regenerative potential of sEVs secreted at distinct stages of myogenic differentiation, we incorporated sEV-ND, sEV-ED, and sEV-LD into collagen scaffolds and implanted these into VML defects in the tibialis anterior (TA) muscle of C57BL/6 mice, and at 2 weeks post-implantation, the tissues were harvested for histological and molecular analyses (Figure 2A). Hematoxylin and eosin (H&E) staining revealed that compared with the muscles treated with bare scaffolds, which were characterized by a disrupted architecture within the defect region, those treated with sEV-loaded scaffolds displayed improved tissue organization (Figure 2B). Furthermore, consistent with active myogenesis, regenerating myofibers with centrally located nuclei were observed more frequently in the sEV-treated groups than in the scaffold-only group, with the sEV-LD group showing the most pronounced histological improvements.[22] In addition, relative to the scaffold-only group, Masson's trichrome (MT) staining indicated reduced collagen deposition in sEV-treated muscles, with the smallest fibrotic area observed in the sEV-LD group (Figure 2B). Consistent with these observations, quantitative analyses based on measurements of the cross-sectional fiber area

Figure 1. Characterization of small extracellular vesicles derived from human skeletal muscle cells during differentiation and analysis of myogenic marker expression following sEV treatment

(A) Phase-contrast images of human skeletal muscle cells (HSkMCs) cultured in differentiation medium at 0, 60, and 120 h. Scale bars, 100 μm. (B) RT-qPCR analysis of *MYF5*, *MYOD1*, and *MYOG* mRNA expression in HSkMCs during differentiation. Data are presented as mean ± SEM, $n = 3$. (C) Transmission electron microscopy (TEM) images of small extracellular vesicles (sEVs) isolated from HSkMCs at different stages: undifferentiated (sEV-ND), early differentiation (sEV-ED), and late differentiation (sEV-LD). Arrowheads indicate vesicles. Scale bars, 100 nm (left) and 50 nm (right). (D) Particle size distribution profiles of sEV-ND, sEV-ED, and sEV-LD measured by DLS. (E) Western blot analysis of CD9, CD63, and CD81 in sEVs. (F) RT-qPCR analysis of *MYF5*, *MYOD1*, and *MYOG* expression in HSkMCs treated with sEV-ND, sEV-ED, or sEV-LD (50 μg/mL and 100 μg/mL). Data are presented as mean ± SEM, $n = 3$. (G) Immunofluorescence images of HSkMCs stained with F-actin (green) and DAPI (blue), and quantification of cell aspect ratio. Data are presented as mean ± SEM, $n = 3$. *$p < 0.05$, **$p < 0.01$, ***$p < 0.001$.

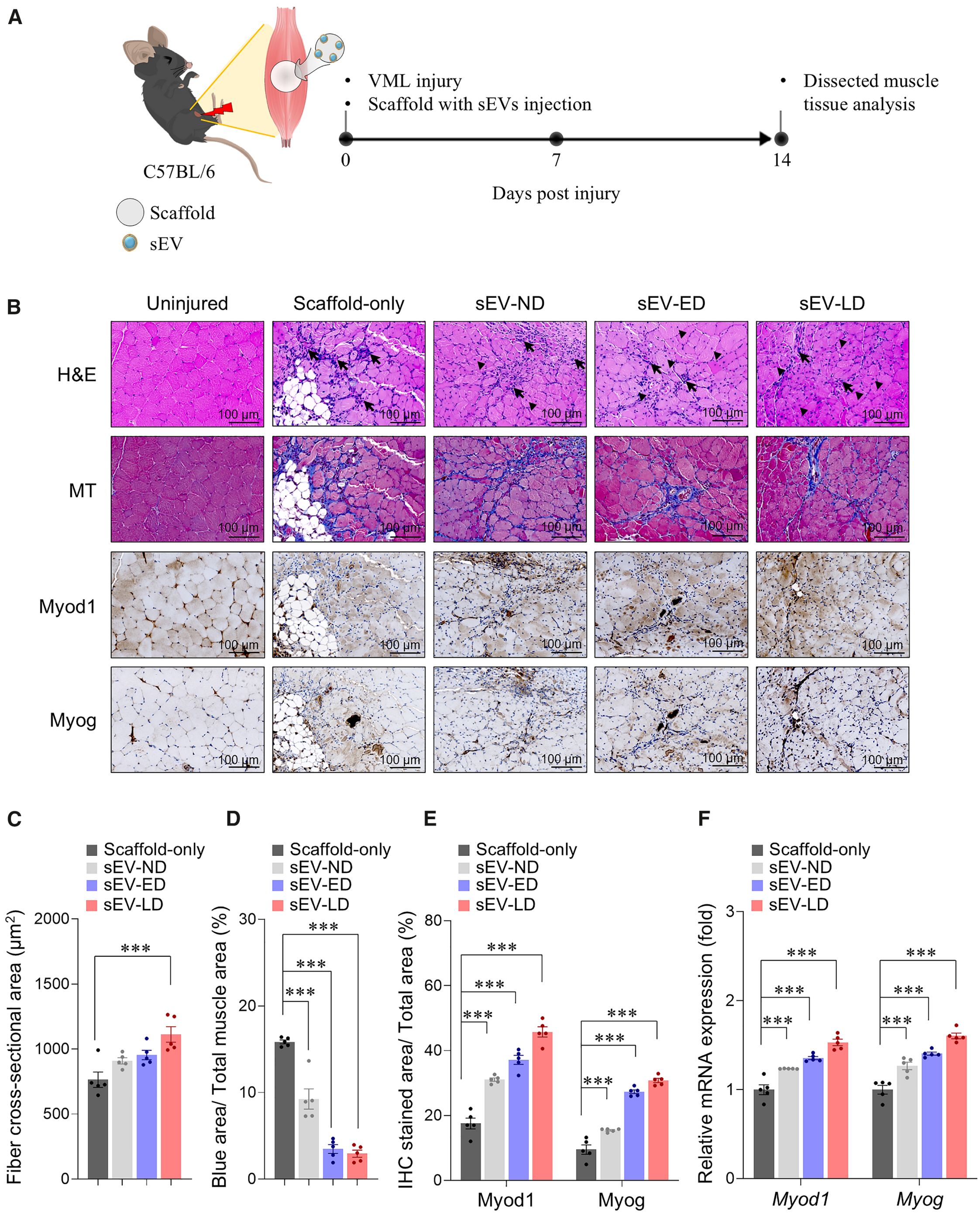

Figure 2. Effects of sEVs derived from human skeletal muscle cells on skeletal muscle tissue in a TA VML model

(A) Schematic illustration of the experimental design showing TA VML injury in C57BL/6 mice and treatment with collagen scaffolds containing sEVs. (B) Histological and immunohistochemical (IHC) images of TA tissues from uninjured, scaffold-only, sEV-ND-, sEV-ED-, and sEV-LD-treated groups. Hematoxylin and eosin (H&E), Masson's trichrome (MT), and IHC staining for Myod1 and Myog are shown. Scale bars, 100 μm. (C) Quantification of fiber cross-sectional area in muscle tissue. (D) Quantification of MT-stained (blue) areas in muscle tissue. (E) Quantification of IHC-positive areas for Myod1 and Myog, expressed as the percentage of stained area relative to total tissue area. (F) RT-qPCR analysis of *Myod1* and *Myog* mRNA expression in TA tissues across treatment groups. Data are presented as mean ± SEM, n = 5 mice per group. *$p < 0.05$, **$p < 0.01$, ***$p < 0.001$. TA, tibialis anterior; VML, volumetric muscle loss; IHC, immunohistochemistry; MT, Masson's trichrome; sEV, small extracellular vesicle; sEV-ND, sEVs from undifferentiated (0 h) HSkMCs; sEV-ED, sEVs from HSkMCs at early differentiation (60 h); sEV-LD, sEVs from HSkMCs at late differentiation (120 h); HSkMC, human skeletal muscle cell.

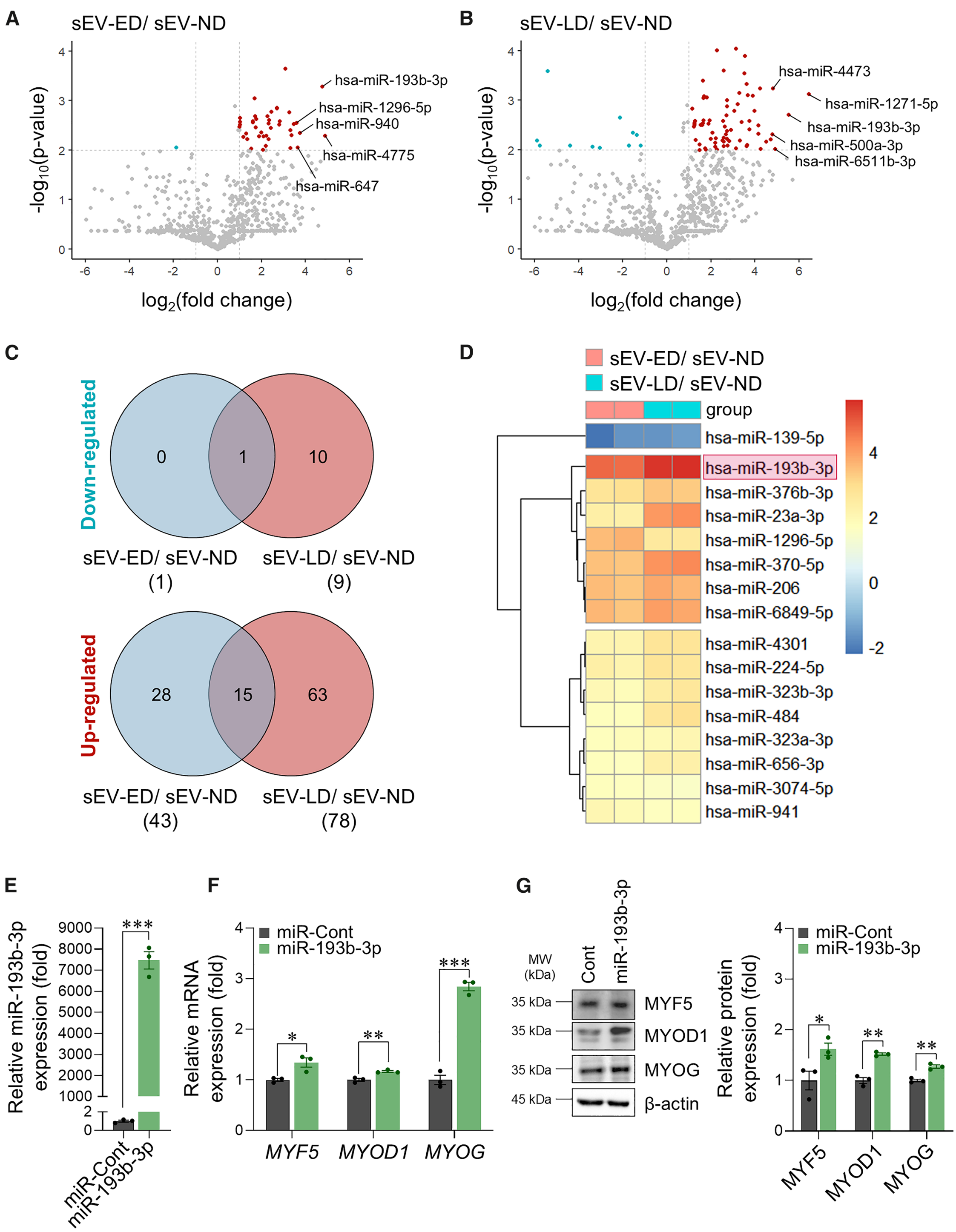

Figure 3. Identification of miR-193b-3p enriched in sEVs during myogenic differentiation and analysis of myogenic marker expression following miR-193b-3p overexpression

(A) Volcano plot of differentially expressed miRNAs (DEMs) in sEV-ED compared to sEV-ND. (B) Volcano plot of DEMs in sEV-LD compared to sEV-ND. (C) Venn diagrams showing the overlap of DEMs between sEV-ED vs. sEV-ND and sEV-LD vs. sEV-ND comparisons. (D) Heatmap of shared DEMs between sEV-ED vs. sEV-ND and sEV-LD vs. sEV-ND comparisons. (E) RT-qPCR analysis of miR-193b-3p expression in HSkMCs transfected with miR-Cont or miR-193b-3p mimic. (F) RT-qPCR analysis of *MYF5*,

(legend continued on next page)

(CFA) revealed an induced increase in myofiber size in the sEV-treated groups, with the most pronounced increase being detected in the sEV-LD group (Figure 2C). In addition, compared with the scaffold-only group, quantitative analysis of MT staining confirmed a marked reduction in fibrotic area in sEV-treated muscles (Figure 2D), whereas in immunohistochemical (IHC) analyses, we observed increases in the staining intensities and distributions of Myod1 and Myog in sEV-treated defects, with the strongest signals again being detected in the sEV-LD group (Figure 2B). For both these markers, quantitative assessments revealed stage-dependent increases in the IHC-positive areas, reaching the highest levels in the sEV-LD group (Figure 2E), and the findings of RT-qPCR analysis similarly indicated a more pronounced upregulation of *Myod1* and *Myog* expression following sEV-LD treatment compared with that observed in response to earlier stage sEVs (Figure 2F). Collectively, these data indicate that differentiation-stage sEVs contribute to enhancing histological and myogenic marker outcomes following volumetric muscle injury, with late-stage sEVs being characterized by the strongest effects. We accordingly speculate that this stage-dependent efficacy may reflect differences in the sEV cargoes associated with myogenic maturation.

miR-193b-3p as a key sEV-associated miRNA enriched during myogenic differentiation that promotes skeletal myogenesis

To identify candidate sEV miRNAs associated with the pro-myogenic effects observed during myogenic differentiation, we performed small RNA sequencing for sEV-ND, sEV-ED, and sEV-LD isolated from HSkMCs. A total of 2,588 miRNAs were detected in sEVs collected at the different stages of myogenic differentiation, among which, we sought to determine those showing differential expression (differentially expressed miRNAs [DEMs]) among groups, defined based on thresholds of a $|\log_2 \text{fold change}| \geq 1$ and p value ≤ 0.01, as illustrated in volcano plots (Figures 3A and 3B) and summarized in Tables S2 and S3. In addition, to identify miRNAs that are dynamically modulated during differentiation, we compared DEMs between sEV-ED and sEV-ND and sEV-LD and sEV-ND, which revealed 16 commonly differentially expressed miRNAs (15 upregulated and one downregulated) (Figure 3C; Table S4). These common miRNAs were further visualized by hierarchical clustering (Figure 3D), among which the upregulated miRNA hsa-miR-193b-3p exhibited the highest fold change in both comparisons and was therefore selected for subsequent functional analysis.

To further assess the potential functional relevance of miR-193b-3p in myogenic differentiation, HSkMCs were transfected with miR-193b-3p mimics, with subsequent RT-qPCR analysis revealing a marked increase in miR-193b-3p expression in the miR-193b-3p-transfected cells compared with those transfected with the miR-Cont sequence (Figure 3E). Functional assays examining myogenic differentiation revealed that the overexpression of miR-193b-3p was associated with increases in the expression of myogenic markers, including *MYF5*, *MYOD1*, and *MYOG* (Figure 3F), with elevations in the corresponding protein levels (Figure 3G). In addition, to assess whether miR-193b-3p contributes to the pro-myogenic effects of sEV-LD, we co-treated HSkMCs with sEV-LD and either inhibitor-miR-Cont or inhibitor-miR-193b-3p, and under these conditions, found that the inhibition of miR-193b-3p was associated with an attenuation of the sEV-LD-induced increases in MYF5, MYOD1, and MYOG protein expression (Figure S2). Collectively, these findings suggest that miR-193b-3p accumulates in differentiation-stage sEVs and may play a role in promoting myogenic differentiation during myogenesis.

AP2M1 as a candidate downstream target of miR-193b-3p

To identify downstream targets via which miR-193b-3p may influence myogenic differentiation, we used three miRNA target prediction algorithms (miRDB, DIANA, and TargetScan). Cross-referencing the outputs from these platforms identified 17 consensus candidate genes (Figure 4A). Among these, WD repeat domain 82 (*WDR82*), adapter-related protein complex 2 subunit mu 1 (*AP2M1*), and tyrosine 3-monooxygenase/tryptophan 5-monooxygenase activation protein zeta (*YWHAZ*) showed reduced mRNA expression following miR-193b-3p mimic transfection in HSkMCs, as assessed by RT-qPCR (Figure 4B). Notably, however, only AP2M1 showed a corresponding reduction at the protein level, indicating that it is a potential downstream target of miR-193b-3p (Figure 4C). To establish whether there is a direct interaction between miR-193b-3p and *AP2M1*, we conducted dual-luciferase reporter assays, which revealed that co-transfection of miR-193b-3p mimics with a reporter construct containing the 3′ untranslated region (3′ UTR) of wild-type *AP2M1* was associated with a reduction in luciferase activity. In contrast, mutating the predicted miR-193b-3p-binding site abolished this effect, thereby providing evidence for a direct interaction between miR-193b-3p and the *AP2M1* 3′ UTR (Figures 4D and 4E). Consistent with these findings, inhibition of endogenous miR-193b-3p was found to result in an increase in AP2M1 protein expression (Figure 4F). To further characterize the potential downstream mechanisms associated with the miR-193b-3p-AP2M1 axis, we examined the signaling pathways associated with receptor trafficking and the regulation of myogenesis, among which, the overexpression of miR-193b-3p was found to lead to a selective reduction in NOTCH1-NTM protein levels, whereas the levels of p-EGFR and β-catenin remained largely unchanged (Figure S3). Consistently, the knockdown of *AP2M1* was similarly found to be associated with a reduction in the expression of

MYOD1, and *MYOG* mRNA expression in HSkMCs following miR-193b-3p overexpression. (G) Western blot analysis of MYF5, MYOD1, and MYOG protein expression in HSkMCs transfected with miR-Cont or miR-193b-3p. Data in (E)–(G) are presented as mean ± SEM, $n = 3$. *$p < 0.05$, **$p < 0.01$, ***$p < 0.001$. HSkMC, human skeletal muscle cell; DEM, differentially expressed miRNA; sEV, small extracellular vesicle; sEV-ND, sEVs from undifferentiated (0 h) HSkMCs; sEV-ED, sEVs from early-stage (60 h) differentiated HSkMCs; sEV-LD, sEVs from late-stage (120 h) differentiated HSkMCs; miR-Cont, negative-control miRNA.

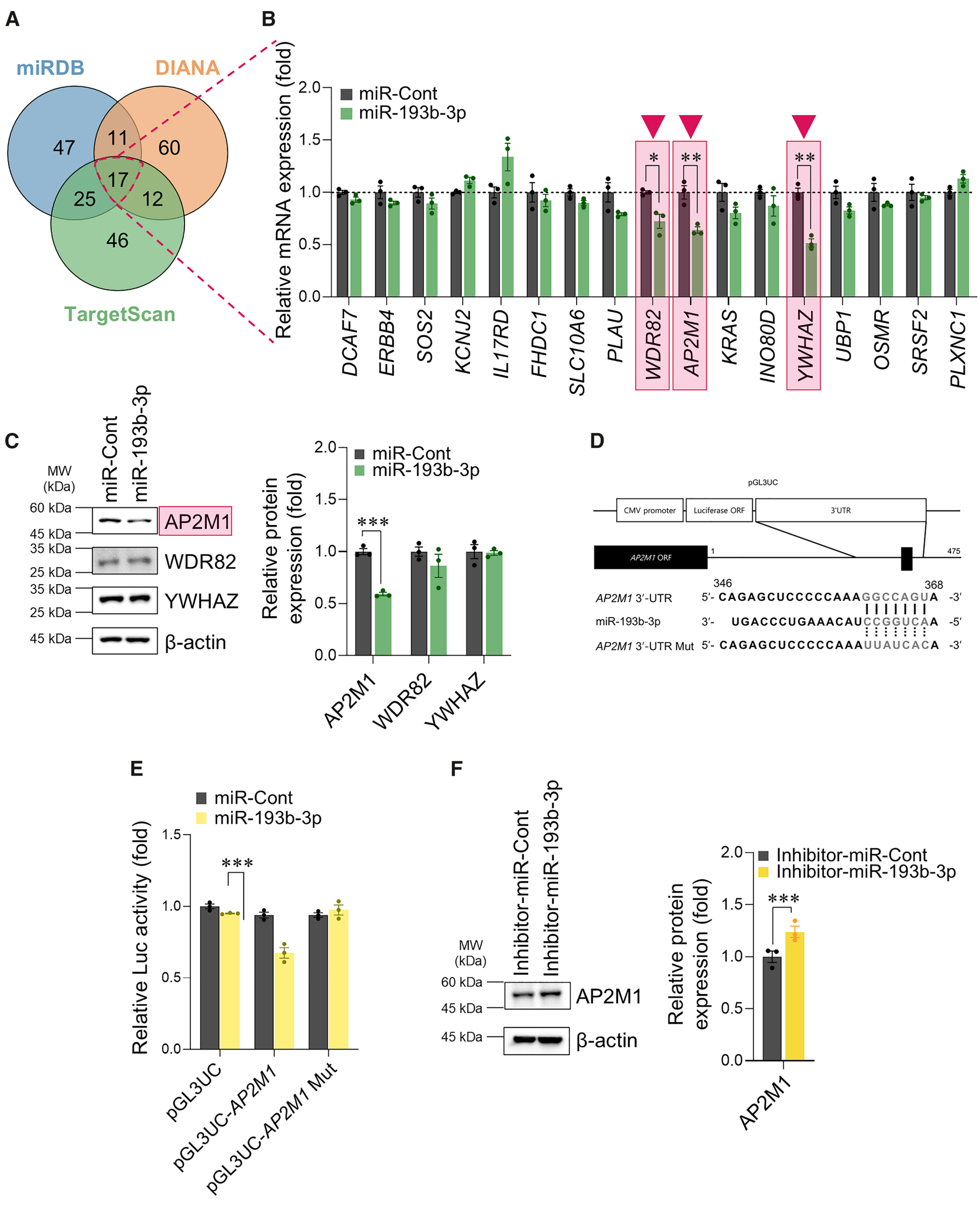

Figure 4. Identification and validation of *AP2M1* as a candidate target of miR-193b-3p

(A) Venn diagram showing the overlap of candidate target genes predicted by miRDB, DIANA, and TargetScan. (B) RT-qPCR analysis of mRNA expression of candidate genes in HSkMCs transfected with miR-Cont or miR-193b-3p. (C) Western blot analysis of AP2M1, WDR82, and YWHAZ protein expression in HSkMCs transfected with miR-Cont or miR-193b-3p. (D) Schematic representation of the *AP2M1* 3′-UTR containing the predicted miR-193b-3p binding site and the corresponding mutant construct. (E) Luciferase reporter assay of pGL3UC (empty vector), pGL3UC-*AP2M1* (WT), and pGL3UC-*AP2M1*-Mut in HSkMCs co-transfected with miR-Cont or miR-193b-3p. (F) Western blot analysis of AP2M1 protein expression in HSkMCs transfected with inhibitor-miR-Cont or inhibitor-miR-193b-3p. Data in (B), (C), (E), and (F) are presented as mean ± SEM, $n = 3$. *$p < 0.05$, **$p < 0.01$, ***$p < 0.001$. HSkMC, human skeletal muscle cell; sEV, small extracellular vesicle; miR-Cont, negative-control miRNA; inhibitor-miR-Cont, control miRNA inhibitor; UTR, untranslated region; WT, wild-type; Mut, mutant.

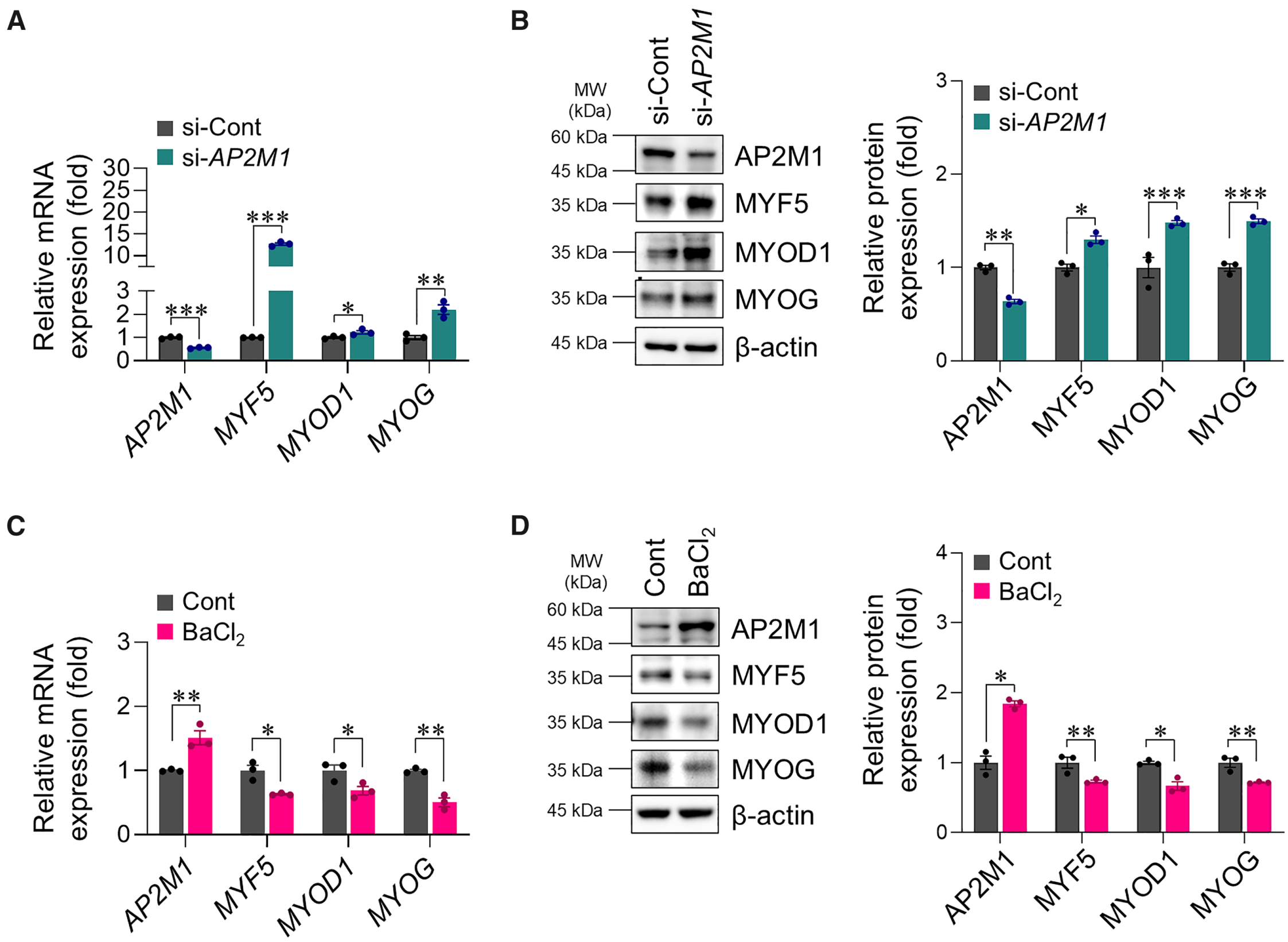

Figure 5. Analysis of AP2M1 and myogenic marker expression under *AP2M1* knockdown and $BaCl_2$ treatment in HSkMCs

(A) RT-qPCR analysis of *AP2M1*, *MYF5*, *MYOD1*, and *MYOG* mRNA expression in HSkMCs transfected with si-Cont or si-*AP2M1*. (B) Western blot analysis of AP2M1, MYF5, MYOD1, and MYOG protein expression in HSkMCs transfected with si-Cont or si-*AP2M1*. (C) RT-qPCR analysis of *AP2M1*, *MYF5*, *MYOD1*, and *MYOG* mRNA expression in HSkMCs treated with $BaCl_2$. (D) Western blot analyses of AP2M1, MYF5, MYOD1, and MYOG protein expression in HSkMCs treated with $BaCl_2$. Data in (A)–(D) are presented as mean ± SEM, $n = 3$. $^*p < 0.05$, $^{**}p < 0.01$, $^{***}p < 0.001$. HSkMC, human skeletal muscle cell; si-Cont, control siRNA; $BaCl_2$, barium chloride.

NOTCH1-NTM, thereby indicating that AP2M1 may influence Notch signaling (Figure S4).

AP2M1 expression is inversely associated with the expression of myogenic differentiation markers

To assess whether the suppression of *AP2M1* is involved in the pro-myogenic effects associated with miR-193b-3p, we silenced *AP2M1* in HSkMCs using siRNA. *AP2M1* knockout was associated with increased expression of the myogenic markers MYF5, MYOD1, and MYOG at both the mRNA and protein levels (Figures 5A and 5B), a pattern similar to that observed following miR-193b-3p overexpression. In addition, to further examine the response of AP2M1 under a mimicked injury condition, we utilized a barium chloride ($BaCl_2$)-induced muscle injury model. HSkMCs exposed to $BaCl_2$ were found to be characterized by higher levels of AP2M1 expression, with concurrent reductions in the expression of myogenic markers (Figures 5C and 5D). Taken together, these findings provide evidence that AP2M1 may serve as a downstream target of miR-193b-3p and function as a negative modulator of myogenic differentiation. Consequently, the miR-193b-3p-AP2M1 axis could represent a regulatory pathway linking differentiation and regeneration during muscle repair.

AP2M1 expression shows increasing age- and sarcopenia-associated trends in human and rodent skeletal muscles

To examine whether *AP2M1* is associated with the degeneration of muscles, we analyzed human and rodent skeletal muscle datasets. In the GEO: GSE111016 human dataset, which includes skeletal muscle samples derived from 20 healthy individuals and 20 patients with sarcopenia, the expression of *AP2M1* was found to be significantly higher in sarcopenic muscles than in those of the healthy controls ($p = 0.024$; Figure 6A). In an independent human dataset, GEO: GSE111006, which includes skeletal muscle samples obtained from 28 healthy individuals and four patients with sarcopenia, *AP2M1* showed a similar upward tendency in sarcopenic samples, although this difference did not reach the level of statistical significance ($p = 0.29$; Figure 6B), which may be attributable to the small sample size of the sarcopenic group. To establish whether this pattern of expression is conserved in other species, we analyzed the GEO: GSE38077 rat dataset profiling tibialis anterior (TA) muscles extracted from young (6-months old) and aged (24-months old) Sprague-Dawley rats. *Ap2m1* expression in aged muscles showed an increase compared with that in young controls, although again this difference was non-significant ($p = 0.22$; Figure 6C). Collectively,

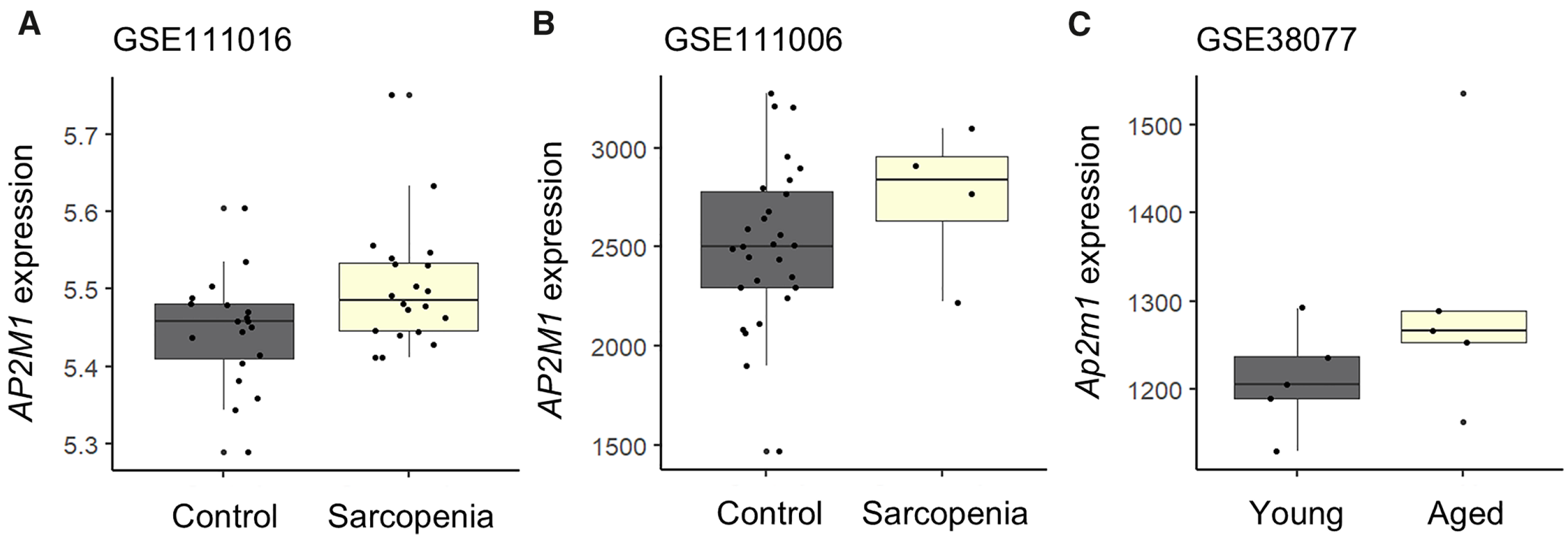

Figure 6. *AP2M1* expression in skeletal muscle across public transcriptomic datasets

(A–C) Boxplots showing *AP2M1* expression in skeletal muscle samples from independent datasets. (A) GEO: GSE111016: control and sarcopenia groups (p = 0.024). (B) GEO: GSE111006: control and sarcopenia groups (p = 0.29). (C) GEO: GSE38077: young and aged groups (p = 0.22). Data are presented as boxplots showing median, interquartile range, and individual data points. GEO, Gene Expression Omnibus; AP2M1, adapter-related protein complex 2 subunit mu 1.

the patterns discerned in these datasets indicate that within the muscles of young and healthy individuals, *AP2M1* tends to be more highly expressed in aged or sarcopenic skeletal muscles in different mammalian species, with statistically significant elevation observed in one human cohort and consistent trends in other datasets. Although not uniformly significant, this pattern indicates that *AP2M1* may be involved in the molecular changes associated with muscle aging and sarcopenia.

miR-193b-3p promotes skeletal muscle regeneration *in vivo*, partly via a suppression of AP2M1

To assess the therapeutic relevance of miR-193b-3p in skeletal muscle repair, we employed a TA VML injury mouse model. Following injury, animals received intramuscular injections of either miR-Cont or miR-193b-3p mimic sequences, and TA muscles were collected 14 days later for histological and molecular analyses (Figure 7A). Compared with those treated with miR-Cont, H&E staining revealed a marked reduction in necrotic muscle fibers and a greater prevalence of regenerating fibers characterized by centrally located nuclei in the miR-193b-3p-treated muscles. In addition, MT staining indicated a reduction in the deposition of collagen in the miR-193b-3p group, with tissue morphology more closely resembling uninjured muscle, whereas more extensive fibrosis was observed in miR-Cont-treated tissues (Figure 7B). These findings were further supported by quantitative analyses. Specifically, measurement of CFA revealed a significant increase in myofiber size in miR-193b-3p-treated muscles compared with miR-Cont-treated muscles (Figure 7C). Additionally, quantitative assessment of MT staining demonstrated a notable reduction in fibrotic area in the miR-193b-3p-treated group relative to the control group (Figure 7D). IHC analysis further revealed that the administration of miR-193b-3p was associated with increases in expression of the myogenic markers Myod1 and Myog, along with reduced expression of its putative downstream target, AP2M1 (Figure 7E), with consistent findings being obtained based on RT-qPCR analyses, revealing upregulation of miR-193b-3p (Figure 7F). In addition, compared with the miR-Cont group, we detected elevated levels of *Myod1* and *Myog* mRNA in the miR-193b-3p group, whereas in contrast, those of *Ap2m1* mRNA were reduced (Figure 7G). Collectively, these data provide *in vivo* support that miR-193b-3p contributes to the regeneration of skeletal muscle following volumetric injury, which is mediated, at least in part, via suppression of AP2M1. These observations are consistent with the proposed functional relevance of the sEV-derived miR-193b-3p-AP2M1 axis in regenerative processes.

DISCUSSION

In this study, we identified a previously understudied sEV-mediated regulatory axis in which miR-193b-3p is associated with skeletal muscle regeneration through the repression of AP2M1, a negative regulator of myogenesis. We found that miR-193b-3p progressively accumulates in sEVs released by differentiating myoblasts, and that these vesicles exert pro-myogenic effects *in vitro* and *in vivo*. These findings suggest an additional layer of temporal regulation during myogenesis and indicate that AP2M1 may represent a potential molecular target for muscle-wasting conditions.

miRNAs are well-established key regulators of muscle development and regeneration, with canonical examples such as miR-1, miR-133, and miR-206 being extensively characterized.[8,9] In recent years, however, attention has shifted to the miR-193 family, with studies reporting that by targeting endocytosis-related genes and signaling pathways, miR-193b has antiproliferative effects in cancer and metabolic disorders.[23,24] Emerging evidence suggests that miR-193b-3p may also play roles in muscle biology. For example, this miRNA has been reported to promote skeletal muscle cell proliferation via IGF2BP1, thereby indicating its potential involvement in muscle regeneration.[25] However, these studies have tended to focus primarily on intracellular interactions, whereas its role in intercellular communication remains unclear. Our findings suggest that sEV-associated miR-193b-3p is upregulated during myogenic differentiation and is associated with increased expression of myogenic markers and morphological changes in myoblasts. Notably, we

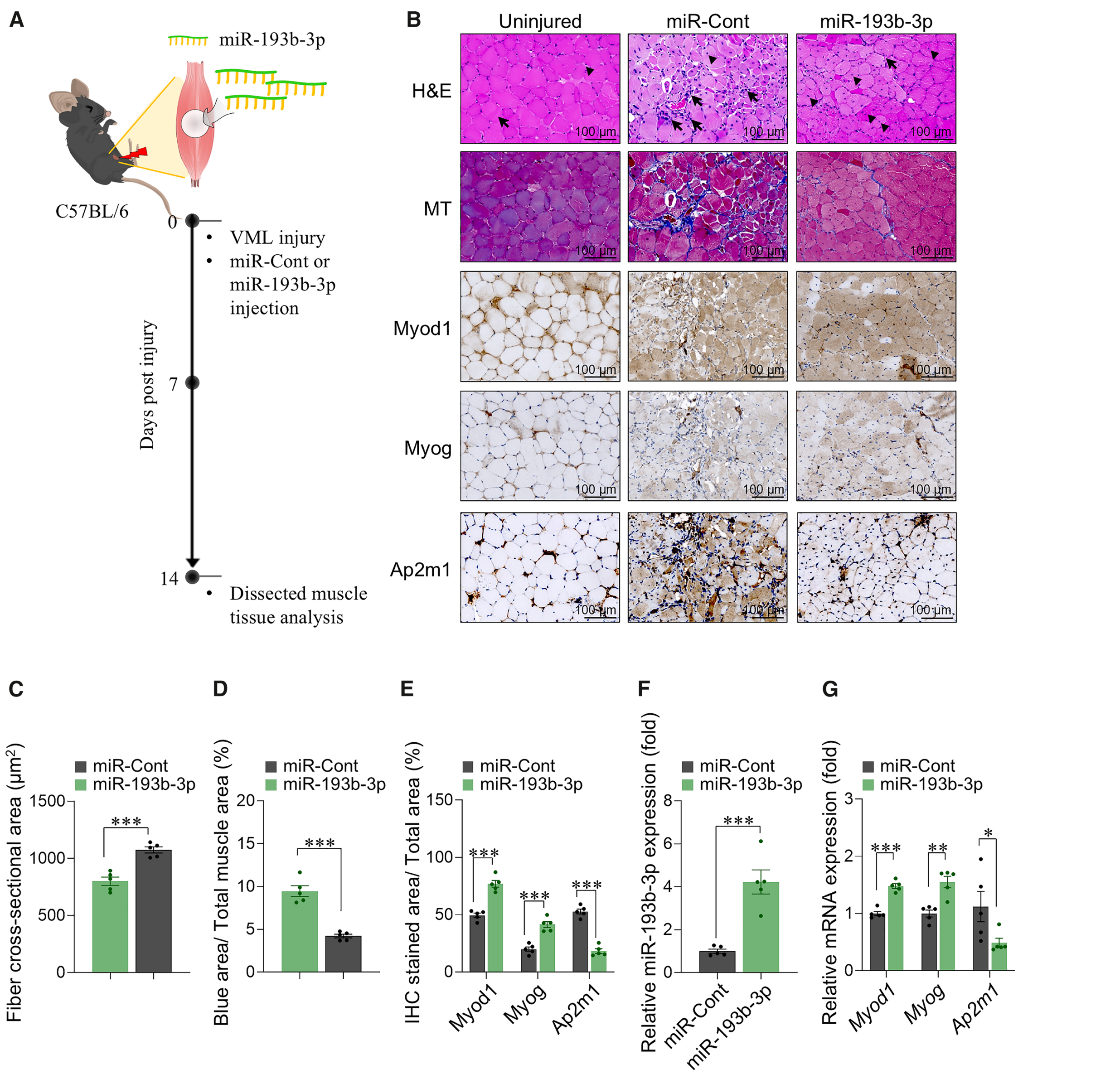

Figure 7. Analysis of histological and molecular features of skeletal muscle following miR-193b-3p treatment in a TA VML model

(A) Schematic representation of the experimental design using a C57BL/6 mouse TA VML injury model with intramuscular injection of miR-Cont or miR-193b-3p and tissue collection at 14 days post-injury. (B) Histological and immunohistochemical images of TA tissues from uninjured, miR-Cont-injected, and miR-193b-3p-injected groups. H&E, MT, and immunohistochemical staining for Myod1, Myog, and Ap2m1 are shown. Scale bars, 100 μm. (C) Quantification of fiber cross-sectional area in TA tissues. (D) Quantification of MT-stained (blue) area in TA tissues. (E) Quantification of IHC-positive areas for Myod1, Myog, and Ap2m1 in TA tissues. (F) RT-qPCR analysis of miR-193b-3p expression in resected TA tissues. (G) RT-qPCR analysis of *Myod1*, *Myog*, and *Ap2m1* mRNA expression in TA tissues. Data in (C)–(G) are presented as mean ± SEM, $n = 5$ mice per group. *$p < 0.05$, **$p < 0.01$, ***$p < 0.001$. TA, tibialis anterior; VML, volumetric muscle loss; IHC, immunohistochemistry; H&E, hematoxylin and eosin; MT, Masson's trichrome.

identified an sEV-mediated miR-193b-3p-AP2M1 axis that may represent a potential mechanistic link between intercellular communication and receptor trafficking.

Contrastingly, some previous studies have reported that under pathological conditions such as type 2 diabetes, miR-193b may impair muscle growth by targeting the PDK1/Akt signaling pathway.[26] This apparent discrepancy may be explained by several factors. First, the miR-193b precursor can undergo strand-specific processing to generate miR-193b-3p and miR-193b-5p, and differences in strand usage may contribute to context-dependent functions in skeletal muscle. Second, differences in experimental contexts, including

pathological versus normal conditions, may influence the functional outcome of miR-193b signaling. Third, whereas previous studies have focused on intracellular functions, we describe an sEV-mediated role of miR-193b-3p, suggesting that its function may vary depending on its mode of delivery and cellular context.

A key finding of this study was our identification of AP2M1, a component of adapter protein complex 2, as a direct target of miR-193b-3p. The AP2 adapter complex consists of α- and β2-adaptins in association with the μ2 and σ2 subunits. *AP2M1* encodes the μ2 subunit, which is essential for clathrin-mediated endocytosis (CME) and the internalization of diverse signaling receptors.[27,28] CME has been established to play a central role in modulating the availability and trafficking of receptors involved in key developmental and regenerative pathways, including Notch, Wnt/β-catenin, and EGFR.[29,30] AP2M1 has also been implicated in neurological processes and disorders, reflecting its key role in receptor trafficking and synaptic function.[31,32] Notch and Wnt/β-catenin signaling, in particular, are well-established regulators of satellite cell activation and postnatal myogenesis.[33] These observations thus suggest that through its role in CME, AP2M1 may contribute to the regulation of signaling pathways essential for muscle regeneration.

Consistent with this, our data suggest that AP2M1 may act as a negative regulator of myogenesis, and that its suppression phenocopies the effects of miR-193b-3p, supporting the involvement of the miR-193b-3p-AP2M1 axis in this process. Given the central role of endocytic trafficking in fine-tuning receptor-mediated signaling, reduced AP2M1 activity may contribute to the modulation of pro-myogenic signaling inputs. In line with this, our experimental data further showed that overexpression of miR-193b-3p and knockdown of AP2M1 reduced NOTCH1-NTM protein levels, suggesting that AP2M1 may modulate Notch signaling.

The *in vivo* relevance of this regulatory circuit was evaluated using a murine VML model. Local administration of miR-193b-3p was associated with enhanced regenerative features, reduced fibrosis, and increased expression of key myogenic transcription factors in regenerating muscle tissues, accompanied by a decrease in AP2M1 levels.[34] These findings suggest that AP2M1 may function as a regulatory modulator that constrains muscle repair and indicate the potential relevance of targeting this pathway.[35,36]

In addition to providing mechanistic insights, our findings may have potential translational relevance. Given that currently adopted interventions for muscle degeneration, including physical therapy, nutritional support, and pharmacological modulation, often yield variable outcomes,[37–39] the therapeutic delivery of miR-193b-3p, potentially in combination with sEV-based approaches, could represent a complementary strategy.[40–42] The observed pro-myogenic effects suggest that miR-193b-3p may have additive or potentially synergistic effects with existing modalities, thereby contributing to a multi-pronged approach to support muscle repair. Future studies are warranted to investigate the applicability of such combined therapeutic strategies in preclinical models and to assess their potential additive or synergistic benefits.[15,38]

To explore the potential relevance of our findings, we analyzed transcriptomic datasets derived from aged rodents and human patients with sarcopenia. Across multiple cohorts, *AP2M1* expression showed an upward trend in degenerative muscle tissues, suggesting a conserved role in age-associated muscle decline. Moreover, these observations further support the potential involvement of the miR-193b-3p-AP2M1 axis in this process.

Despite its strengths, this study has several limitations. First, the upstream regulatory mechanisms controlling miR-193b-3p expression during satellite cell activation, as well as the downstream signaling pathways influenced by *AP2M1* repression, remain to be fully elucidated. In addition, the complexity of sEV cargo should be considered, as other RNA species, including mRNAs, long non-coding RNAs, and additional candidate miRNAs, may also contribute to the observed biological effects, and the relative contribution of miR-193b-3p within this network remains to be defined. In this study, we focused on miRNAs due to their well-established roles in regulating gene expression during myogenesis and their ability to be directly linked to downstream targets and functional outcomes. Importantly, the *in vivo* sEV and miR-193b-3p experiments were designed to address sequential rather than comparative questions. The former established the stage-dependent regenerative activity of differentiation-derived sEVs, whereas the latter examined the functional relevance of miR-193b-3p identified from their cargo. Therefore, these experiments should not be interpreted as a direct comparison of regenerative efficacy between whole sEVs and miR-193b-3p. Rather, our findings indicate that miR-193b-3p represents one functionally relevant component contributing to the pro-myogenic activity of differentiation-derived sEVs, while the overall regenerative effects of sEVs likely reflect the combined actions of multiple bioactive cargos. However, although treatment with sEVs and miR-193b-3p increased the expression of myogenic markers *in vivo*, the magnitude of this effect was relatively modest. Therefore, further studies are needed to enhance the *in vivo* efficacy of these approaches and clarify their functional significance. Moreover, while histological and molecular markers of regeneration were assessed, long-term functional evaluations of muscle recovery, such as muscle strength or endurance, were not included and warrant future investigation.

In summary, we identified an sEV-mediated regulatory axis in which miR-193b-3p is associated with skeletal muscle regeneration via repression of *AP2M1*. Our findings contribute to an improved understanding of sEV-mediated intercellular communication during myogenesis and suggest that the miR-193b-3p-AP2M1 interaction may represent a potential target for muscle-wasting conditions. The enrichment of miR-193b-3p in sEVs derived from differentiating myoblasts, together with its pro-myogenic effects observed *in vitro* and *in vivo*, supports its potential functional relevance. Collectively, these findings provide a basis for further investigation

into miRNA- and sEV-based approaches for skeletal muscle repair. In particular, sEV-mediated delivery of regenerative miRNAs may provide a basis for developing therapeutic strategies for promoting muscle regeneration in conditions such as traumatic muscle injury or muscle-wasting disorders.

MATERIALS AND METHODS

Cells

Primary human skeletal muscle cells (HSkMCs: PCS-950–010; ATCC) were obtained from the American Type Culture Collection (ATCC) and cultured in mesenchymal stem cell basal medium supplemented with a Primary Skeletal Muscle Growth Kit (PCS-950–040; ATCC) and 1% penicillin-streptomycin (15140122; Gibco) at 37°C in a humidified 5% CO_2 incubator. Myogenesis was induced by seeding 2×10^5 HSkMCs per well in six-well plates, and culturing them in skeletal muscle DM (Skeletal Muscle Differentiation Tool, PCS-950–050; ATCC) for 60 or 120 h, with medium changes at 2- to 3-day intervals.[43]

sEV isolation

sEVs were isolated from HSkMCs using ExoQuick-TC (EXOTC50A-1; System Biosciences), according to the manufacturer's protocol. Briefly, 5×10^6 HSkMCs were seeded in 150-mm dishes and incubated in growth medium or DM. sEVs were collected at defined time points corresponding to the growth conditions: sEV-ND, 60 h in growth medium; sEV-ED, 0–60 h in DM; and sEV-LD, 60–120 h in DM. The total sEV yield at each time point was quantified using a BCA protein assay. At the indicated time points, culture supernatants were collected and centrifuged at 3,000 rpm for 30 min to remove cellular debris. The clarified supernatants were mixed with ExoQuick-TC in a 1:5 ratio, incubated overnight at 4°C, and then centrifuged at 3,000 rpm for 30 min. The supernatants were discarded after centrifugation, and the sEV pellets were resuspended in 1× phosphate-buffered saline (PBS), stored at −80°C, and used within 1 month. The protein concentrations of HSkMC-derived sEVs were determined using a Pierce BCA Protein Assay Kit (23225; Thermo Fisher Scientific) according to the manufacturer's instructions. For subsequent experiments, sEV preparations were normalized to give comparable stock concentrations (1 mg/mL), equal amounts of which (50 or 100 μg/mL) were used for treatment.

Morphology and size distribution of sEVs

sEV morphology and size distribution were analyzed using TEM (JEM 1011 transmission electron microscope; JEOL) and DLS (ELSZ-2000ZS; Otsuka Electronics). For TEM, the sEVs were adsorbed onto formvar/carbon-coated mesh grids, negatively stained with 2% uranyl acetate, and imaged. For DLS, sEV suspensions were diluted to 1 μg/mL in PBS and measured in duplicate after temperature stabilization. Raw DLS intensity data were processed using R (version 4.3.0) with the ggplot2, dplyr, purrr, and gridExtra packages. To exclude interference from large aggregates and debris, particle size distributions were restricted to a range of 30–1,000 nm to exclude large aggregates and debris, fitted using a log-normal model, and normalized as intensity (%).[44] Modal diameters (mode) were calculated and expressed as the mean ± SEM of two independent replicates.

Western blot analysis of sEV surface markers

Western blot analysis was performed to detect surface marker proteins on sEVs. Proteins were extracted from sEVs using PRO-PREP Protein Extraction Solution (17081; iNtRON Biotechnology). Equal amounts of protein (20 μg) were separated on 8% SDS-PAGE gels at 100 V for 1 h and transferred onto PVDF membranes at 95 V for 1 h. After blocking with 5% skimmed milk in TBST for 1 h at 25°C, the membranes were incubated overnight at 4°C with the following primary antibodies: CD9 (1:1,000: sc-13118; Santa Cruz), CD63 (1:1,000: sc-5275, Santa Cruz), CD81 (1:1,000: MA5-13548; Invitrogen), and β-actin (1:2,000: sc-47778; Santa Cruz). After washing, the membranes were incubated for 2 h at 25°C with horseradish peroxidase (HRP)-conjugated secondary antibodies (anti-mouse IgG or anti-rabbit IgG [both 1:4,000: ACE BioLabs]). Protein bands were visualized using an EZ-Western Lumi Pico kit (DG-WP; DoGENBio).

Ethical statement and animals

This study complied with all relevant ethical guidelines for animal research and was conducted in accordance with the ARRIVE guidelines and the NIH Guide for the Care and Use of Laboratory Animals. All animal procedures were approved by the Institutional Animal Care and Use Committee of the CHA Laboratory Animal Research Center (approval no. #200076). Male C57BL/6N mice were purchased from KOATECH and maintained under specific pathogen-free conditions at 23°C ± 1°C and 50% ± 10% humidity, with a 12-h light/dark cycle. Food and water were provided ad libitum.

Volumetric TA muscle loss injury model

To assess the therapeutic effects of sEVs and miRNA mimics on skeletal muscle regeneration, we performed two independent VML injury experiments using the TA muscles of mice. In both experiments, a full-thickness muscle defect (3 mm in diameter and 2 mm in depth) was created using a biopsy punch after fur removal and an anterior incision under anesthesia. Each experimental group consisted of five mice. After treatment, the skin was sutured and 2 weeks later, TA muscle samples were harvested for regeneration analysis.[45] The mice were euthanized via CO_2 inhalation at 30% volume/min flow rate and death was confirmed by the absence of breathing and eye discoloration.

sEV treatment

To evaluate the regenerative potential of HSkMC-derived sEVs during myogenic differentiation, mice were divided into two groups: (1) the sEVs group, in which mice received a collagen sponge loaded with 100 μg sEV suspended in 10 μL of PBS and implanted into the VML site, and (2) the Sham group, in which the mice received a collagen sponge without sEVs implanted into the corresponding defect site. The sEVs used for group 1 were classified into three types according to the stage of HSkMC differentiation: (1) sEV-ND; (2)

sEV-ED; and (3) sEV-LD. The surgical site was sutured following implantation, and the mice were euthanized 2 weeks later, with TA muscle tissues being collected for assessments of regeneration.

miRNA mimic treatment

To assess the effects of miR-193b-3p on muscle regeneration, mice were divided into two groups: (1) the miR-193b-3p mimic group, in which mice were intramuscularly injected with 10 μg of miR-193b-3p mimic complexed with 1.2 μL of in vivo-jetPEI transfection reagent (201-50G; Polyplus-transfection SA) in a final volume of 50 μL, and (2) the negative control group, in which mice received the same amount of control mimic mixed with transfection reagent using the same protocol. Prior to injecting into the VML site, the mixture was incubated for 15 min at 25°C. The mice were euthanized 2 weeks after treatment and TA muscle tissues were harvested for assessments of regeneration.

Histological and immunohistochemical analysis

TA muscle tissues were resected and fixed in 10% formalin for 24 h at 4°C for subsequent histological and immunohistochemical analyses. Tissues were embedded in paraffin, sectioned at 5 μm thickness, deparaffinized with xylene, and rehydrated using a graded ethanol series. H&E and MT staining were performed for histological evaluation. For IHC analysis, sections were deparaffinized, rehydrated, and subjected to antigen retrieval by microwaving using Antigen Retrieval Buffer (ab93678; Abcam) for 15 min. Endogenous peroxidase activity was inhibited by treatment with 3% hydrogen peroxide for 30 min at 25°C, followed by a 15-min incubation in 3% bovine serum albumin at 25°C. Thereafter, the slides were incubated overnight at 4°C with primary antibodies against Myod1 (1:100: MA5-12902; Thermo Fisher Scientific), Myog (1:100: CMA5-11486, Thermo Fisher Scientific), and Ap2m1 (1:100: NBP2-67762; Novus Biologicals), and then treated with HRP-conjugated anti-mouse secondary antibody (1:200: A1012S; ACE Biolabs) or anti-rabbit secondary antibody (1:200: A1013S; ACE Biolabs) for 20 min at 25°C. After washing with PBS, immunoreactivity was visualized using 3,3′-diaminobenzidine (DAB) chromogen for 10 min at 25°C. Images were acquired using a ZEISS Axioscan 7 microscope in bright-field scanning mode (Zeiss GmbH).

RT-qPCR

Total RNA was extracted from HSkMCs and dissected TA muscle tissues using TRIzol reagent (15596018; Thermo Fisher Scientific) according to the manufacturer's instructions. For mRNA and miRNA expression analysis, 1 μg total RNA was reverse-transcribed to complementary DNA (cDNA) using a Reverse Transcription Master Mix (EBT-1542; ELPIS-BIOTECH) for mRNA or an HB miR Multi Assay Kit (SYSTEM I; HeimBiotek) for miRNA. The reverse transcription conditions were 42°C for 30 min followed by 94°C for 5 min for mRNA synthesis, and 37°C for 1 h followed by 95°C for 5 min for miRNA synthesis. RT-qPCRs for mRNA and miRNA were conducted using an EzAmp qPCR 2× Master Mix (EBT-1801; ELPIS-BIOTECH) for mRNA or an HB SYSTEM I miR Multi Assay Kit for miRNA, following the respective protocols. Thermocycling conditions for mRNA quantification included an initial denaturation at 95°C for 3 min, followed by 40 cycles at 95°C for 10 s, 60°C for 10 s, and 72°C for 30 s. For miRNA detection, the conditions included an initial denaturation step at 95°C for 15 min, followed by 40 cycles of 95°C for 5 s and 60°C for 40 s. The expression of mRNAs was normalized to that of the *GAPDH* internal control, and miRNA levels were normalized to those of RNU6B. All experiments were performed in triplicate. Relative expression values were calculated using the $2^{-\Delta\Delta Cq}$ method.[46] Details regarding the sequences of primers used for amplification are shown in Table S1.

Western blot analysis of cellular proteins

Western blotting for HSkMCs was conducted using the same protocol described in "Western blot analysis of sEV surface markers" section. Briefly, cells were either transfected with RNA oligonucleotides or subjected to $BaCl_2$-induced injury, harvested in PBS, and lysed using a PRO-PREP Protein Extraction Solution. Equal amounts of protein (20 μg) were loaded onto 10% SDS-PAGE gels, followed by PVDF membrane transfer and blocking with 5% skimmed milk. Membranes were incubated overnight at 4°C with primary antibodies against MYF5 (1:1,000: sc-518039; Santa Cruz), MYOD1 (1:1,000), MYOG (1:500), AP2M1 (1:1,000), and β-actin (1:2,000: sc-47778; Santa Cruz). After washing, HRP-conjugated secondary antibodies were applied, and the bands were detected as described in Section above.

Small miRNA sequencing and differentially expressed miRNA analysis

To profile the miRNA cargoes in sEVs, we sequenced small RNAs. Sequencing was conducted commercially by E-Biogen Inc. (Seoul, Republic of Korea) to analyze DEMs. Libraries were prepared using an NEBNext Multiplex Small RNA Library Prep Kit (New England BioLabs, MA, USA) according to the manufacturer's protocol. RNA samples were ligated to adapters, reverse-transcribed using adapter-specific primers, and PCR amplified. The PCR products were purified using a QIAquick PCR Purification Kit (QIAGEN, Düsseldorf, Germany) and AMPure XP beads (Beckman Coulter, Pasadena, CA, USA). Library quality and fragment size distribution were assessed using an Agilent 2100 Bioanalyzer in conjunction with a high-sensitivity DNA assay (Agilent Technologies). High-throughput sequencing was performed using the Illumina NextSeq500 platform with single-end 75 bp reads (Illumina, San Diego, CA, USA). Sequencing reads were aligned to mature miRNA reference sequences from miRBase (v.22.1) using Bowtie2 (v.2.4.2) and read counts were quantified using BEDTools (v.2.25.0) and the Bioconductor packages miRBase.db (v.1.2.0) and miRNA (v.1.58.0) in R (v.3.2.2; R Foundation for Statistical Computing, Vienna, Austria). Analysis of differential expression was performed between groups, with miRNAs showing a $|\log_2(\text{fold-change})| \geq 1$ and a p value <0.01 being defined as DEMs. Visualization was carried out in R using the ggplot2, VennDiagram, and pheatmap packages, and volcano plots were generated to illustrate the distribution of DEMs. Venn diagrams were used to identify overlapping and unique

DEMs between comparisons, and hierarchical clustering heat maps were constructed following log transformation and normalization of the expression values. Clustering was performed using correlation-based distances with complete linkages. For candidate screening, DEMs were ranked according to their statistical significance and magnitude of fold-change, with the top-ranked miRNAs being selected for downstream experimental validation.

Transfection with RNA oligonucleotides

For transfection experiments, we used a range of RNA oligonucleotides, including miRNA mimics, inhibitors, and siRNAs. An miR-193b-3p mimic with the sequence 5′–AACUGGCCCUCAAAGUCC CGCU–3′ (obtained from the miRBase database [version 22.1; https://www.mirbase.org]) was synthesized as an RNA duplex by Genolution. The control miRNA (miR-Cont) sequence, similarly synthesized by Genolution, was 5′–CCUCGUGCCGUUCCAUCAGGUA GUU–3′. miRNA inhibitors were synthesized by BIONICS as 2′-*O*-methyl-modified oligoribonucleotide single strands, with the following sequences: inhibitor-miR-193b-3p (5′–AGCGGGACUUU GAGGGCCAGUU–3′) and inhibitor-miR-Cont, (5′–CAGUACUU UUGUGUAGUACAA–3′). Small interfering RNAs (siRNAs) targeting *AP2M1* (si-*AP2M1*; sc-60184), *WDR82* (si-*WDR82*: sc-78161), and *YWHAQ* (si-*YWHAQ*: sc-29586), as well as control siRNA (si-Cont: sc-37007), were purchased from Santa Cruz Biotechnology. For transfection, RNA oligonucleotides were combined with the G-fectin transfection reagent (Genolution) to give a final concentration of 40 nM, according to the manufacturer's protocol. The transfection mixture was added to 5×10^4 HSkMCs per well seeded in six-well plates and incubated at 37°C for 48 h.

miRNA target gene selection

Putative target genes of miR-193b-3p were predicted using selected miRNA target prediction tools, namely, miRDB version 6.0 (https://www.mirdb.org), TargetScan release 8.0 (https://www.targetscan.org), and DIANA-MICROT version 4 (https://dianalab.e-ce.uth.gr/html/universe/index.php?r=microtv4). Candidate genes were selected based on their identification as common targets using all three prediction algorithms.

Luciferase assays

The potential binding sites of miR-193b-3p within the 3′ untranslated region (3′ UTR) of the *AP2M1* gene were predicted using the TargetScan tool (https://www.targetscan.org). Fragments of the *AP2M1* 3′ UTR containing the predicted binding sites were cloned into a pGL3UC vector by Macrogen Inc. (provided by Kim, Seoul National University, Seoul, South Korea). To generate mutant reporters, nucleotide sequences lacking the miR-193b-3p-binding site were synthesized and inserted into the pGL3UC vector by Macrogen. HSkMCs (5×10^4 cells per well) were seeded in 24-well plates, incubated for 24 h, and then co-transfected with 100 ng reporter plasmid or empty pGL3UC, 100 ng *Renilla* luciferase plasmid, and 40 nM miR-Cont or miR-193b-3p using Lipofectamine 3000 (L3000008; Thermo Fisher Scientific). After a 48-h incubation at 37°C, the cells were lysed using 1× Passive Lysis Buffer (E1910, Promega Corporation) to release the Firefly and *Renilla* luciferase enzymes. The luciferase activity of both the Firefly and *Renilla* enzymes was measured using a Dual-Luciferase Reporter Assay System (E1910, Promega Corporation) following the manufacturer's instructions. Firefly luciferase activity was normalized to that of *Renilla* luciferase.

$BaCl_2$-induced *in vitro* muscle injury model

To establish an *in vitro* model of muscle injury, we used $BaCl_2$, which has been widely applied in both *in vivo* and *in vitro* skeletal muscle injury models,[47,48] as a chemical stimulus that induces membrane disruption and cytotoxic effects in muscle cells. HSkMCs (2×10^5 cells per well) were seeded in six-well plates and cultured with the DM for 48 h to induce myogenic differentiation. Following differentiation, the medium was replaced with fresh growth medium containing 0.012% $BaCl_2$ (202738, Sigma-Aldrich) and incubated for 3 h at 37°C to induce chemical injury. At the indicated time points after injury, the cells were harvested for protein and RNA extraction for western blot and RT-qPCR analyses, respectively. Control cells were treated under the same conditions without exposure to $BaCl_2$.

Transcriptomic analysis of *AP2M1* expression in rodent and human skeletal muscles

To evaluate the pattern of *AP2M1* expression in skeletal muscle, we retrieved transcriptomic datasets from the Gene Expression Omnibus (GEO) database. For the rodent model, we analyzed the GSE38077 dataset, which contains gene expression profiles from the TA muscles of 6- and 24-month-old Sprague-Dawley rats ($n = 5$ per group), whereas for human skeletal muscle, we obtained two independent GEO datasets, namely, GSE111006 (skeletal muscle biopsies from 28 healthy individuals and four patients with sarcopenia) and GSE111016 (20 healthy and 20 sarcopenic muscle samples). Normalized expression data were obtained from the GEO database. Boxplots were generated using R (v.3.6.0) with the ggplot2 package, and statistical differences between groups were assessed using the Student's t test.

Statistical analysis

Statistical analyses were performed using GraphPad Prism Software 8 (Dotmatics). Differences between two groups were evaluated using unpaired Student's t tests, whereas comparisons among more than two groups were assessed using a one-way analysis of variance (ANOVA), followed by Tukey's post-hoc test. Data are presented as the means ± standard error of the mean (SEM), with statistical significance being defined as a p value <0.05.

DATA AND CODE AVAILABILITY

All data generated or analyzed during this study are included in this published article and the supplementary information files. Additional raw data are available from the corresponding authors on reasonable request.

ACKNOWLEDGMENTS

This work was supported by National Research Foundation of Korea (NRF) grants funded by the Korean government (MSIT) (no. RS-2023–00210067 and RS-2022-NR070174). All animal procedures were approved by the IACUC of the CHA Laboratory

Animal Research Center (approval no. #2000076). This study complied with all relevant institutional and international guidelines for animal research. All procedures were performed in accordance with the ARRIVE guidelines and the NIH Guide for the Care and Use of Laboratory Animals. Consent to participate is not applicable because this study did not involve human subjects.

AUTHOR CONTRIBUTIONS

S.C., investigation, data curation, visualization, and writing – original draft; M.S., formal analysis and visualization; H.-J.A., conceptualization, funding acquisition, project administration, writing – review and editing, and methodology; S.L., conceptualization, funding acquisition, project administration, writing – review and editing, and formal analysis. All authors have read and approved the final manuscript.

DECLARATION OF INTERESTS

Some authors are inventors on a patent application related to this work.

DECLARATION OF GENERATIVE AI AND AI-ASSISTED TECHNOLOGIES IN THE WRITING PROCESS

During the preparation of this work the author(s) used AI (ChatGPT) for language editing. After using this tool/service, the authors reviewed and edited the content as needed and take full responsibility for the content of the publication.

SUPPLEMENTAL INFORMATION

Supplemental information can be found online at https://doi.org/10.1016/j.omtn.2026.103087.

REFERENCES

1. Dumont, N.A., Bentzinger, C.F., Sincennes, M.C., and Rudnicki, M.A. (2015). Satellite Cells and Skeletal Muscle Regeneration. Compr. Physiol. *5*, 1027–1059. https://doi.org/10.1002/cphy.c140068.
2. Relaix, F., and Zammit, P.S. (2012). Satellite cells are essential for skeletal muscle regeneration: the cell on the edge returns centre stage. Development *139*, 2845–2856. https://doi.org/10.1242/dev.069088.
3. Hawke, T.J., and Garry, D.J. (2001). Myogenic satellite cells: physiology to molecular biology. J. Appl. Physiol. *91*, 534–551. https://doi.org/10.1152/jappl.2001.91.2.534.
4. Langridge, B., Griffin, M., and Butler, P.E. (2021). Regenerative medicine for skeletal muscle loss: a review of current tissue engineering approaches. J. Mater. Sci. Mater. Med. *32*, 15. https://doi.org/10.1007/s10856-020-06476-5.
5. Muñoz-Cánoves, P., Neves, J., and Sousa-Victor, P. (2020). Understanding muscle regenerative decline with aging: new approaches to bring back youthfulness to aged stem cells. FEBS J. *287*, 406–416. https://doi.org/10.1111/febs.15182.
6. Huo, F., Liu, Q., and Liu, H. (2022). Contribution of muscle satellite cells to sarcopenia. Front. Physiol. *13*, 892749. https://doi.org/10.3389/fphys.2022.892749.
7. Singh, G.B., Cowan, D.B., and Wang, D.Z. (2020). Tiny Regulators of Massive Tissue: MicroRNAs in Skeletal Muscle Development, Myopathies, and Cancer Cachexia. Front. Oncol. *10*, 598964. https://doi.org/10.3389/fonc.2020.598964.
8. Chen, J.F., Mandel, E.M., Thomson, J.M., Wu, Q., Callis, T.E., Hammond, S.M., Conlon, F.L., and Wang, D.Z. (2006). The role of microRNA-1 and microRNA-133 in skeletal muscle proliferation and differentiation. Nat. Genet. *38*, 228–233. https://doi.org/10.1038/ng1725.
9. Mitchelson, K.R., and Qin, W.Y. (2015). Roles of the canonical myomiRs miR-1, -133 and -206 in cell development and disease. World J. Biol. Chem. *6*, 162–208. https://doi.org/10.4331/wjbc.v6.i3.162.
10. Wang, J., Yang, L.Z., Zhang, J.S., Gong, J.X., Wang, Y.H., Zhang, C.L., Chen, H., and Fang, X.T. (2018). Effects of microRNAs on skeletal muscle development. Gene *668*, 107–113. https://doi.org/10.1016/j.gene.2018.05.039.
11. Liu, N., Williams, A.H., Maxeiner, J.M., Bezprozvannaya, S., Shelton, J.M., Richardson, J.A., Bassel-Duby, R., and Olson, E.N. (2012). microRNA-206 promotes skeletal muscle regeneration and delays progression of Duchenne muscular dystrophy in mice. J. Clin. Investig. *122*, 2054–2065. https://doi.org/10.1172/JCI62656.
12. Qi, W., and Guan, W. (2024). A Comprehensive Review on the Importance of MiRNA-206 in the Animal Model and Human Diseases. Curr. Neuropharmacol. *22*, 1064–1079. https://doi.org/10.2174/1570159X21666230407124146.
13. Fochi, S., Giuriato, G., De Simone, T., Gomez-Lira, M., Tamburin, S., Del Piccolo, L., Schena, F., Venturelli, M., and Romanelli, M.G. (2020). Regulation of microRNAs in Satellite Cell Renewal, Muscle Function, Sarcopenia and the Role of Exercise. Int. J. Mol. Sci. *21*, 6732. https://doi.org/10.3390/ijms21186732.
14. Mytidou, C., Koutsoulidou, A., Zachariou, M., Prokopi, M., Kapnisis, K., Spyrou, G.M., Anayiotos, A., and Phylactou, L.A. (2021). Age-Related Exosomal and Endogenous Expression Patterns of miR-1, miR-133a, miR-133b, and miR-206 in Skeletal Muscles. Front. Physiol. *12*, 708278. https://doi.org/10.3389/fphys.2021.708278.
15. Porcu, C., Dobrowolny, G., and Scicchitano, B.M. (2024). Exploring the Role of Extracellular Vesicles in Skeletal Muscle Regeneration. Int. J. Mol. Sci. *25*, 5811. https://doi.org/10.3390/ijms25115811.
16. Ferguson, S.W., Wang, J., Lee, C.J., Liu, M., Neelamegham, S., Canty, J.M., and Nguyen, J. (2018). The microRNA regulatory landscape of MSC-derived exosomes: a systems view. Sci. Rep. *8*, 1419. https://doi.org/10.1038/s41598-018-19581-x.
17. Li, S., Lv, D., Yang, H., Lu, Y., and Jia, Y. (2023). A review on the current literature regarding the value of exosome miRNAs in various diseases. Ann. Med. *55*, 2232993. https://doi.org/10.1080/07853890.2023.2232993.
18. Nakamura, Y., Miyaki, S., Ishitobi, H., Matsuyama, S., Nakasa, T., Kamei, N., Akimoto, T., Higashi, Y., and Ochi, M. (2015). Mesenchymal-stem-cell-derived exosomes accelerate skeletal muscle regeneration. FEBS Lett. *589*, 1257–1265. https://doi.org/10.1016/j.febslet.2015.03.031.
19. Jia, J., Wang, L., Zhou, Y., Zhang, P., and Chen, X. (2024). Muscle-derived extracellular vesicles mediate crosstalk between skeletal muscle and other organs. Front. Physiol. *15*, 1501957. https://doi.org/10.3389/fphys.2024.1501957.
20. Shao, X., Gong, W., Wang, Q., Wang, P., Shi, T., Mahmut, A., Qin, J., Yao, Y., Yan, W., Chen, D., et al. (2022). Atrophic skeletal muscle fibre-derived small extracellular vesicle miR-690 inhibits satellite cell differentiation during ageing. J. Cachexia Sarcopenia Muscle *13*, 3163–3180. https://doi.org/10.1002/jcsm.13106.
21. Cao, X., Xue, L., Yu, X., Yan, Y., Lu, J., Luo, X., Wang, H., and Wang, J. (2024). Myogenic exosome miR-140-5p modulates skeletal muscle regeneration and injury repair by regulating muscle satellite cells. Aging (Albany NY) *16*, 4609–4630. https://doi.org/10.18632/aging.205617.
22. Zheng, L., Liu, X., Chen, P., and Xiao, W. (2019). Expression and role of lncRNAs in the regeneration of skeletal muscle following contusion injury. Exp. Ther. Med. *18*, 2617–2627. https://doi.org/10.3892/etm.2019.7871.
23. Sun, L., He, M., Xu, N., Xu, D.H., Ben-David, Y., Yang, Z.Y., and Li, Y.J. (2018). Regulation of RAB22A by mir-193b inhibits breast cancer growth and metastasis mediated by exosomes. Int. J. Oncol. *53*, 2705–2714. https://doi.org/10.3892/ijo.2018.4571.
24. Yu, H., Peng, Y., Wu, Z., Wang, M., and Jiang, X. (2022). MiR-193b as an effective biomarker in human cancer prognosis for Asian patients: a meta-analysis. Transl. Cancer Res. *11*, 2249–2261. https://doi.org/10.21037/tcr-21-2557.
25. Li, L., Zhang, X., Yang, H., Xu, X., Chen, Y., Dai, D., Zhan, S., Guo, J., Zhong, T., Wang, L., et al. (2022). miR-193b-3p Promotes Proliferation of Goat Skeletal Muscle Satellite Cells through Activating IGF2BP1. Int. J. Mol. Sci. *23*, 15760. https://doi.org/10.3390/ijms232415760.
26. Yang, S., Yang, G., Wu, H., Kang, L., Xiang, J., Zheng, P., Qiu, S., Liang, Z., Lu, Y., and Jia, L. (2022). MicroRNA-193b impairs muscle growth in mouse models of type 2 diabetes by targeting the PDK1/Akt signalling pathway. Diabetologia *65*, 563–581. https://doi.org/10.1007/s00125-021-05616-y.
27. Tsai, K.W., Leung, C.M., Lo, Y.H., Chen, T.W., Chan, W.C., Yu, S.Y., Tu, Y.T., Lam, H.C., Li, S.C., Ger, L.P., et al. (2016). Arm Selection Preference of MicroRNA-193a Varies in Breast Cancer. Sci. Rep. *6*, 28176. https://doi.org/10.1038/srep28176.
28. Mettlen, M., Chen, P.H., Srinivasan, S., Danuser, G., and Schmid, S.L. (2018). Regulation of Clathrin-Mediated Endocytosis. Annu. Rev. Biochem. *87*, 871–896. https://doi.org/10.1146/annurev-biochem-062917-012644.
29. Sigismund, S., Argenzio, E., Tosoni, D., Cavallaro, E., Polo, S., and Di Fiore, P.P. (2008). Clathrin-mediated internalization is essential for sustained EGFR signaling

but dispensable for degradation. Dev. Cell *15*, 209–219. https://doi.org/10.1016/j.devcel.2008.06.012.

30. Andersson, E.R. (2012). The role of endocytosis in activating and regulating signal transduction. Cell. Mol. Life Sci. *69*, 1755–1771. https://doi.org/10.1007/s00018-011-0877-1.

31. Helbig, I., Lopez-Hernandez, T., Shor, O., Galer, P., Ganesan, S., Pendziwiat, M., Rademacher, A., Ellis, C.A., Hümpfer, N., Schwarz, N., et al. (2019). A Recurrent Missense Variant in AP2M1 Impairs Clathrin-Mediated Endocytosis and Causes Developmental and Epileptic Encephalopathy. Am. J. Hum. Genet. *104*, 1060–1072. https://doi.org/10.1016/j.ajhg.2019.04.001.

32. Gear, R., Kalitsis, P., Glass, M., Isidor, B., Vincent-Delorme, C., Petit, F., Verhagen, J.M.A., Jorge, A., Krepischi, A.C.V., Osei-Owusu, I., et al. (2025). AP2M1 Is a Candidate Gene for Microcephaly and Intellectual Disability in 3q27.1 Deletions. Am. J. Med. Genet. *197*, e64153. https://doi.org/10.1002/ajmg.a.64153.

33. Tsivitse, S. (2010). Notch and Wnt signaling, physiological stimuli and postnatal myogenesis. Int. J. Biol. Sci. *6*, 268–281. https://doi.org/10.7150/ijbs.6.268.

34. Yoshimoto, Y., Ikemoto-Uezumi, M., Hitachi, K., Fukada, S.I., and Uezumi, A. (2020). Methods for Accurate Assessment of Myofiber Maturity During Skeletal Muscle Regeneration. Front. Cell Dev. Biol. *8*, 267. https://doi.org/10.3389/fcell.2020.00267.

35. Zimmerman, S.P., DeGraw, L.B., and Counter, C.M. (2025). The essential clathrin adapter protein complex-2 is tumor suppressive specifically in vivo. Nat. Commun. *16*, 2254. https://doi.org/10.1038/s41467-025-57521-2.

36. Groot, M., and Lee, H. (2020). Sorting Mechanisms for MicroRNAs into Extracellular Vesicles and Their Associated Diseases. Cells *9*, 1044. https://doi.org/10.3390/cells9041044.

37. Csala, D., Ádám, Z., and Wilhelm, M. (2025). The Role of miRNAs and Extracellular Vesicles in Adaptation After Resistance Exercise: A Review. Curr. Issues Mol. Biol. *47*, 583. https://doi.org/10.3390/cimb47080583.

38. Wan, R., Liu, S., Feng, X., Luo, W., Zhang, H., Wu, Y., Chen, S., and Shang, X. (2024). The Revolution of exosomes: From biological functions to therapeutic applications in skeletal muscle diseases. J. Orthop. Translat. *45*, 132–139. https://doi.org/10.1016/j.jot.2024.01.001.

39. Luo, J., Pu, Q., and Wu, X. (2024). Recent Advances of Exosomes Derived from Skeletal Muscle and Crosstalk with Other Tissues. Int. J. Mol. Sci. *25*, 10877. https://doi.org/10.3390/ijms252010877.

40. Yedigaryan, L., Martínez-Sarrà, E., Giacomazzi, G., Giarratana, N., van der Veer, B.K., Rotini, A., Querceto, S., Grosemans, H., Cortés-Calabuig, Á., Salucci, S., et al. (2022). Extracellular vesicle-derived miRNAs improve stem cell-based therapeutic approaches in muscle wasting conditions. Front. Immunol. *13*, 977617. https://doi.org/10.3389/fimmu.2022.977617.

41. Mazzucchelli, L., Sarcon, A.K., Huang, T.C.T., Li, J., Berry, C.E., Houdek, M.T., Behfar, A., Zhao, C., and Moran, S.L. (2023). A Ready-to-Use Purified Exosome Product for Volumetric Muscle Loss and Functional Recovery. Tissue Eng. Part A *29*, 481–490. https://doi.org/10.1089/ten.TEA.2023.0057.

42. Zhang, X., Zhang, D., Zhang, Y., Wang, J., and Lu, J. (2025). Restoration of skeletal muscle function via mesenchymal stem cells: mechanistic insights and therapeutic advances in myasthenia gravis. Front. Cell Dev. Biol. *13*, 1658062. https://doi.org/10.3389/fcell.2025.1658062.

43. Noone, J., Rochfort, K.D., O'Sullivan, F., and O'Gorman, D.J. (2023). SIRT4 is a regulator of human skeletal muscle fatty acid metabolism influencing inner and outer mitochondrial membrane-mediated fusion. Cell. Signal. *112*, 110931. https://doi.org/10.1016/j.cellsig.2023.110931.

44. Gandham, S., Su, X., Wood, J., Nocera, A.L., Alli, S.C., Milane, L., Zimmerman, A., Amiji, M., and Ivanov, A.R. (2020). Technologies and Standardization in Research on Extracellular Vesicles. Trends Biotechnol. *38*, 1066–1098. https://doi.org/10.1016/j.tibtech.2020.05.012.

45. Larouche, J.A., Wallace, E.C., Spence, B.D., Buras, E., and Aguilar, C.A. (2023). Spatiotemporal mapping of immune and stem cell dysregulation after volumetric muscle loss. JCI Insight *8*, e162835. https://doi.org/10.1172/jci.insight.162835.

46. Livak, K.J., and Schmittgen, T.D. (2001). Analysis of relative gene expression data using real-time quantitative PCR and the 2(-Delta Delta C(T)) Method. Methods *25*, 402–408. https://doi.org/10.1006/meth.2001.1262.

47. Fleming, J.W., Capel, A.J., Rimington, R.P., Player, D.J., Stolzing, A., and Lewis, M.P. (2019). Functional regeneration of tissue engineered skeletal muscle in vitro is dependent on the inclusion of basement membrane proteins. Cytoskeleton (Hoboken) *76*, 371–382. https://doi.org/10.1002/cm.21553.

48. Hardy, D., Besnard, A., Latil, M., Jouvion, G., Briand, D., Thépenier, C., Pascal, Q., Guguin, A., Gayraud-Morel, B., Cavaillon, J.M., et al. (2016). Comparative Study of Injury Models for Studying Muscle Regeneration in Mice. PLoS One *11*, e0147198. https://doi.org/10.1371/journal.pone.0147198.

OMTN, Volume *37*

Supplemental information

Myogenic differentiation-derived small extracellular vesicles as a therapeutic strategy for miR-193b-3p-mediated muscle regeneration

Sujin Choi, Min-Ji Sung, Hyun-Ju An, and Soonchul Lee

Table S1. Primers used in this study (provided as a separate Excel file).

Table S2. List of DEMs in sEV-ED compared with sEV-ND (provided as a separate Excel file).

Table S3. List of DEMs in sEV-LD compared with sEV-ND (provided as a separate Excel file).

Table S4. The common list of DEMs between sEV-ED vs. sEV-ND and sEV-LD vs. sEV-ND.

Gene symbol	Fold change		p-value	
	sEV-ED/ sEV-ND	sEV-LD/ sEV-ND	sEV-ED/ sEV-ND	sEV-LD/ sEV-ND
hsa-miR-193b-3p	26.7408	45.5644	0.0005	0.0019
hsa-miR-23a-3p	4.7919	17.6417	0.0021	0.0047
hsa-miR-370-5p	9.9262	17.5993	0.0040	0.0032
hsa-miR-6849-5p	9.9387	13.2359	0.0090	0.0038
hsa-miR-206	10.2581	12.2363	0.0050	0.0083
hsa-miR-376b-3p	6.9722	9.7388	0.0030	0.0007
hsa-miR-484	3.2981	6.6408	0.0034	0.0082
hsa-miR-224-5p	4.6629	6.5526	0.0051	0.0013
hsa-miR-4301	4.2666	6.4162	0.0053	0.0020
hsa-miR-1296-5p	11.9674	5.9637	0.0028	0.0003
hsa-miR-323b-3p	3.6983	5.8763	0.0052	0.0068
hsa-miR-656-3p	3.2233	4.7527	0.0009	0.0001
hsa-miR-323a-3p	3.3752	3.9308	0.0023	0.0059
hsa-miR-941	3.9232	3.1788	0.0044	0.0008
hsa-miR-3074-5p	3.2300	2.8548	0.0020	0.0031
hsa-miR-139-5p	0.2696	0.3427	0.0088	0.0045

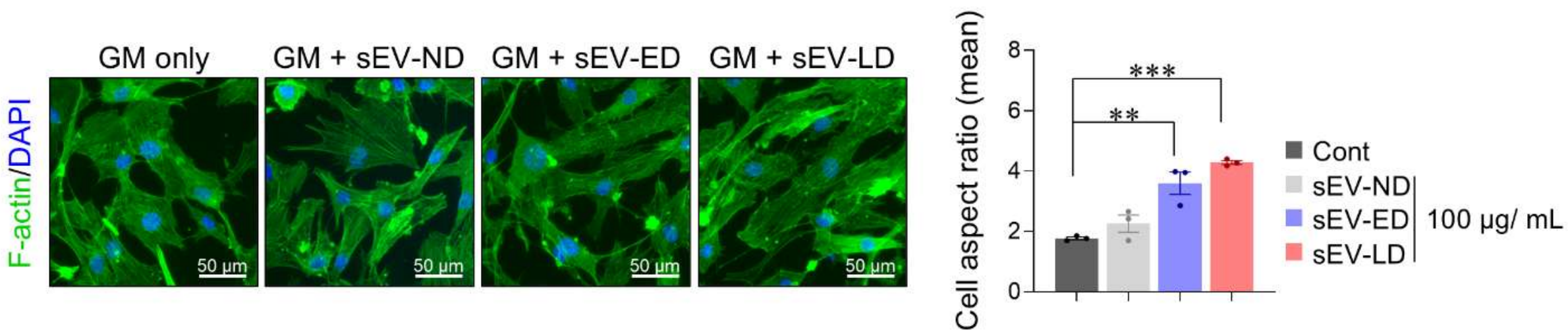

Figure S1. Immunofluorescence images of HSkMCs stained with F-actin (green) and DAPI (blue), and quantification of cell aspect ratio under growth medium conditions with sEV treatment.

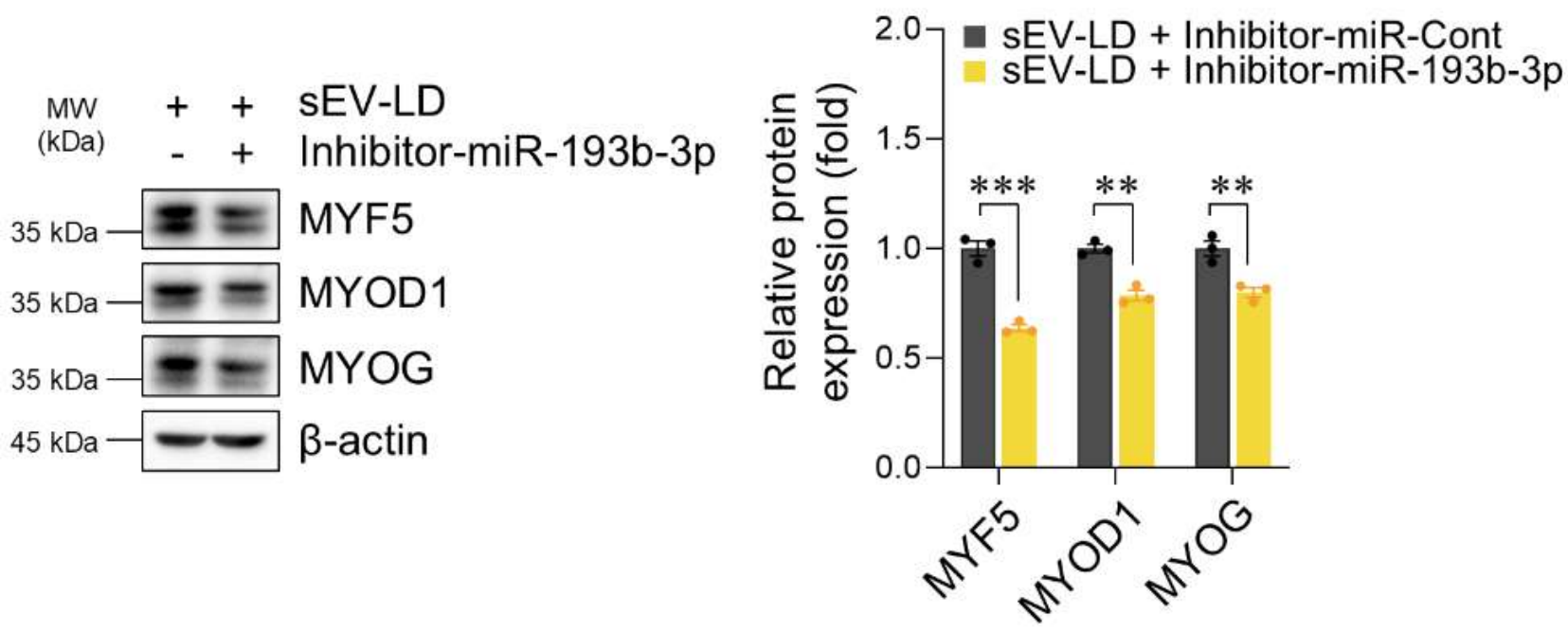

Figure S2. Western blot analysis of MYF5, MYOD1, and MYOG in HSkMCs co-treated with sEV-LD and either inhibitor-miR-Cont or inhibitor-miR-193b-3p.

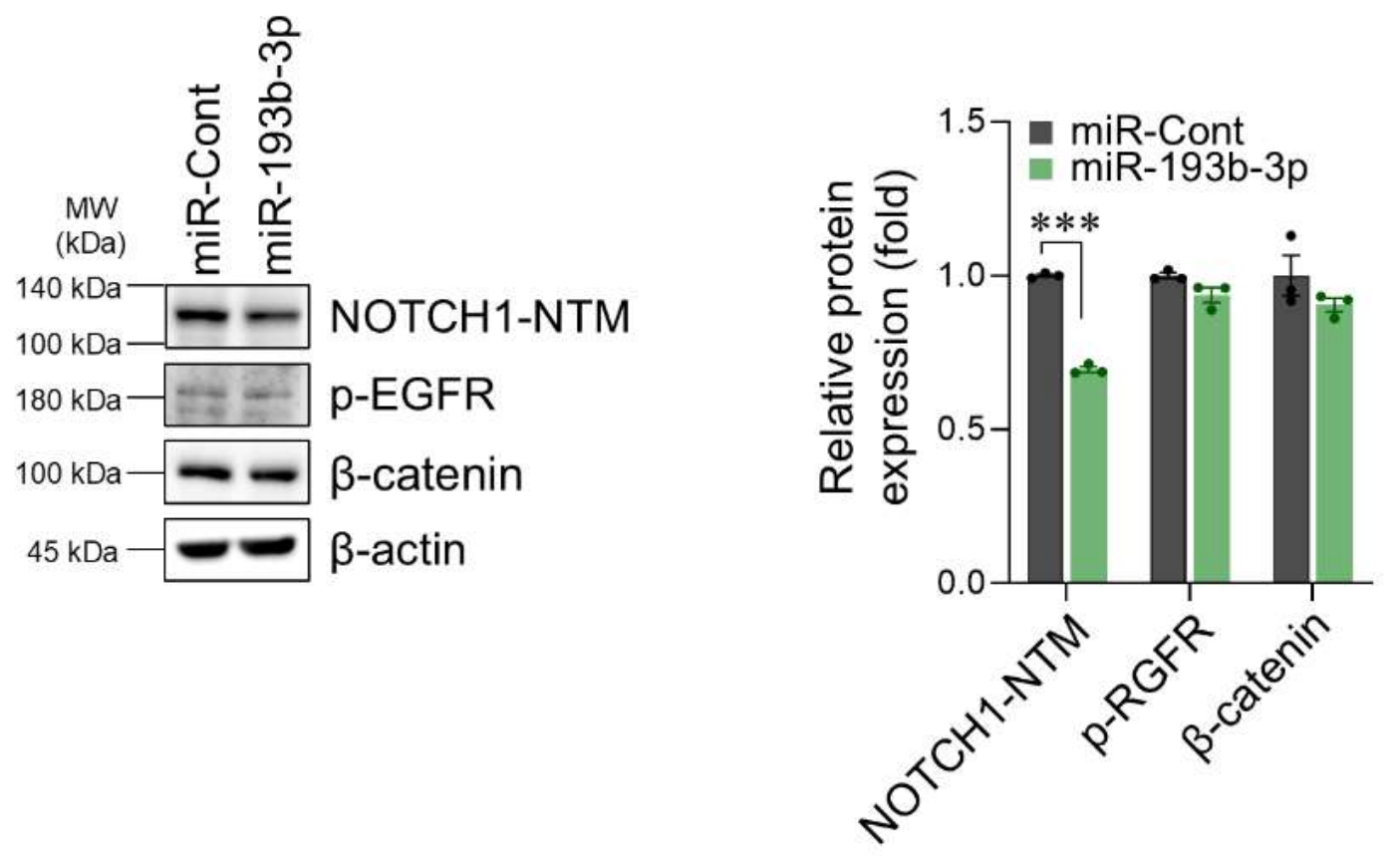

Figure S3. Western blot analysis of NOTCH1-NTM, p-EGFR, and β-catenin in HSkMCs transfected with either miR-Cont or miR-193b-3p.

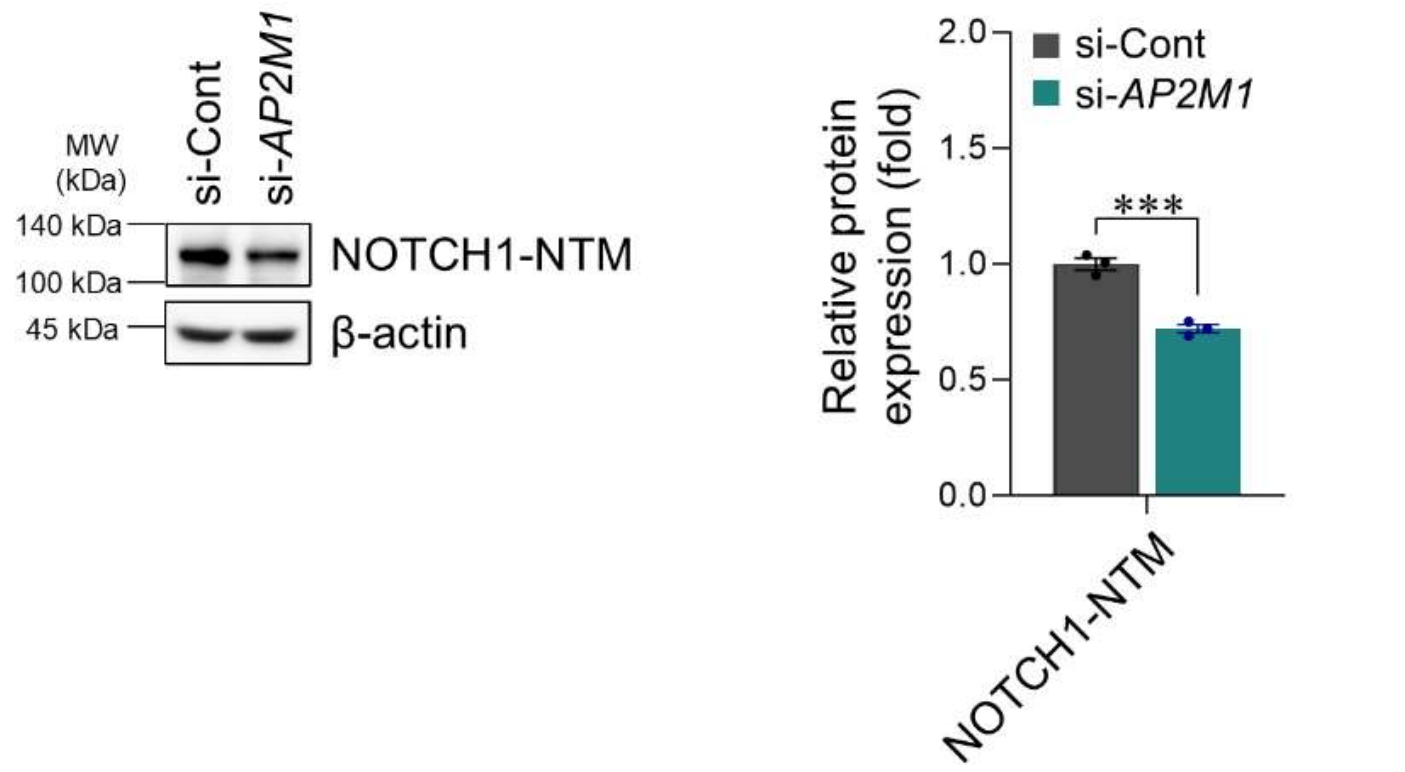

Figure S4. Western blot analysis of NOTCH1-NTM in HSkMCs transfected with either si-Cont or si-*AP2M1*.

A

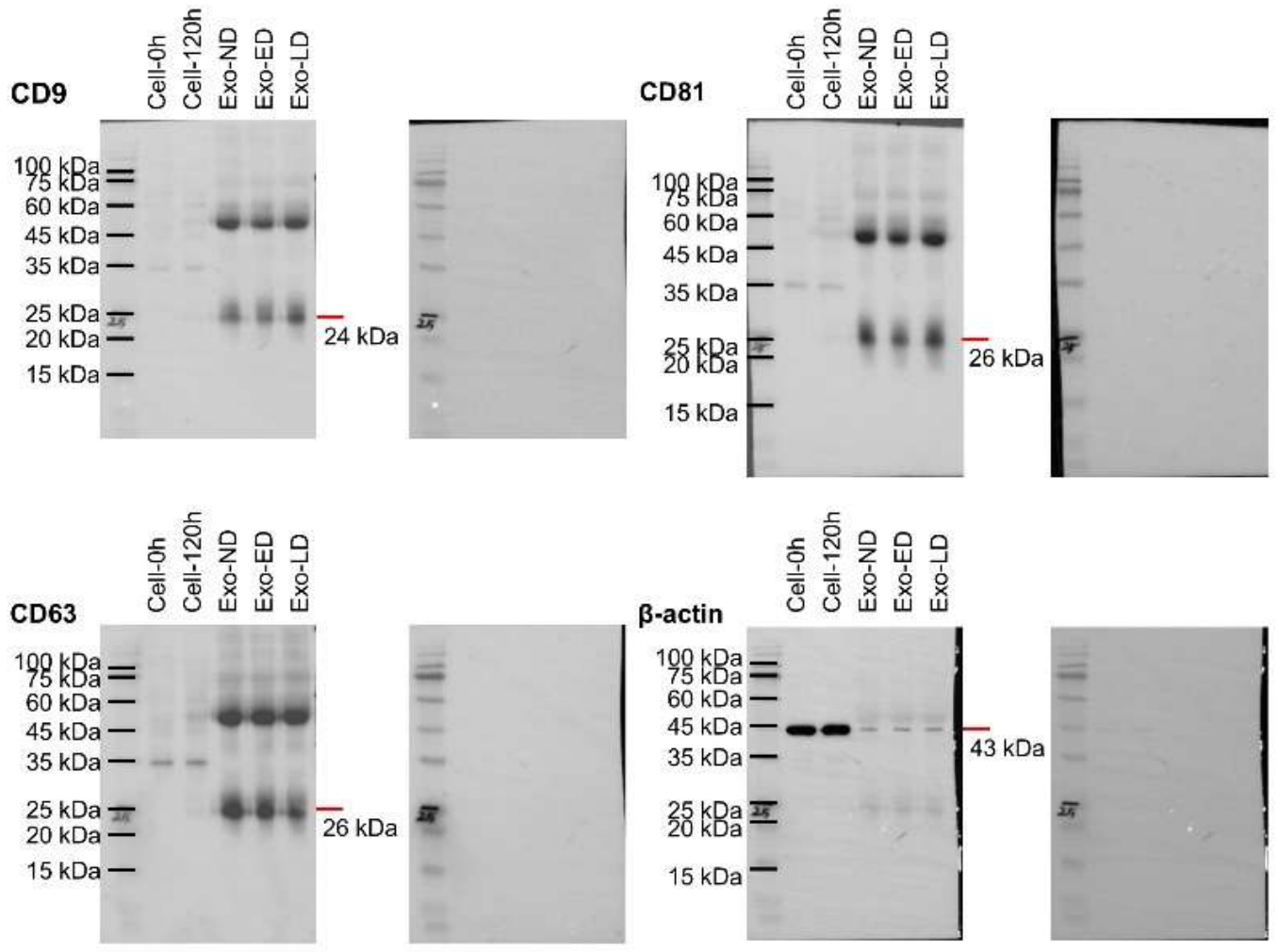
CD9
Cell-0h
Cell-120h
Exo-ND
Exo-ED
Exo-LD
100 kDa
75 kDa
60 kDa
45 kDa
35 kDa
25 kDa
20 kDa
15 kDa
24 kDa
CD81
26 kDa
CD63
26 kDa
β-actin
43 kDa

B

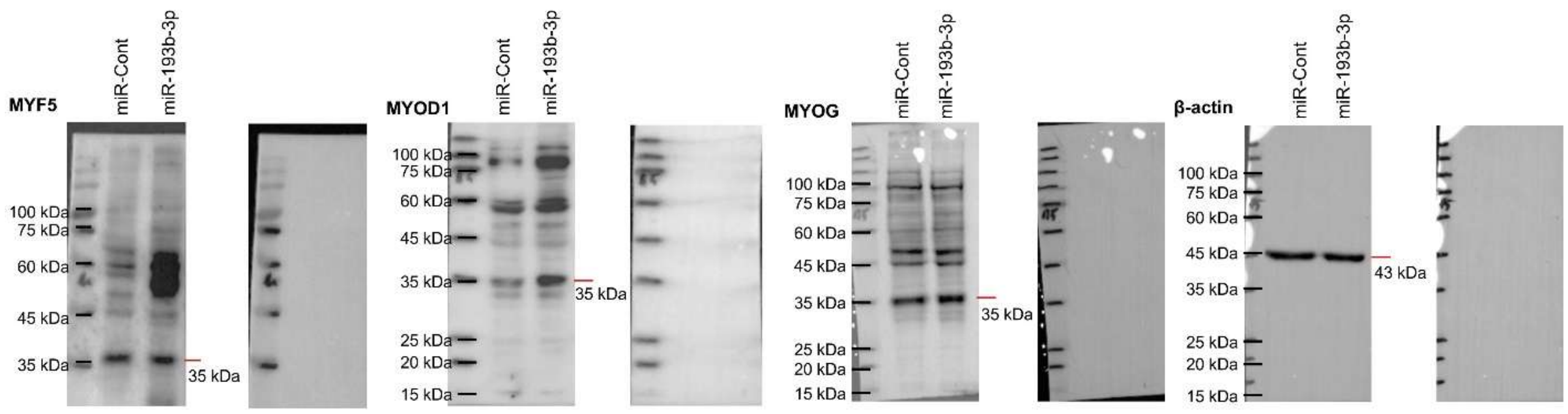
MYF5
miR-Cont
miR-193b-3p
35 kDa
MYOD1
35 kDa
MYOG
35 kDa
β-actin
43 kDa

C

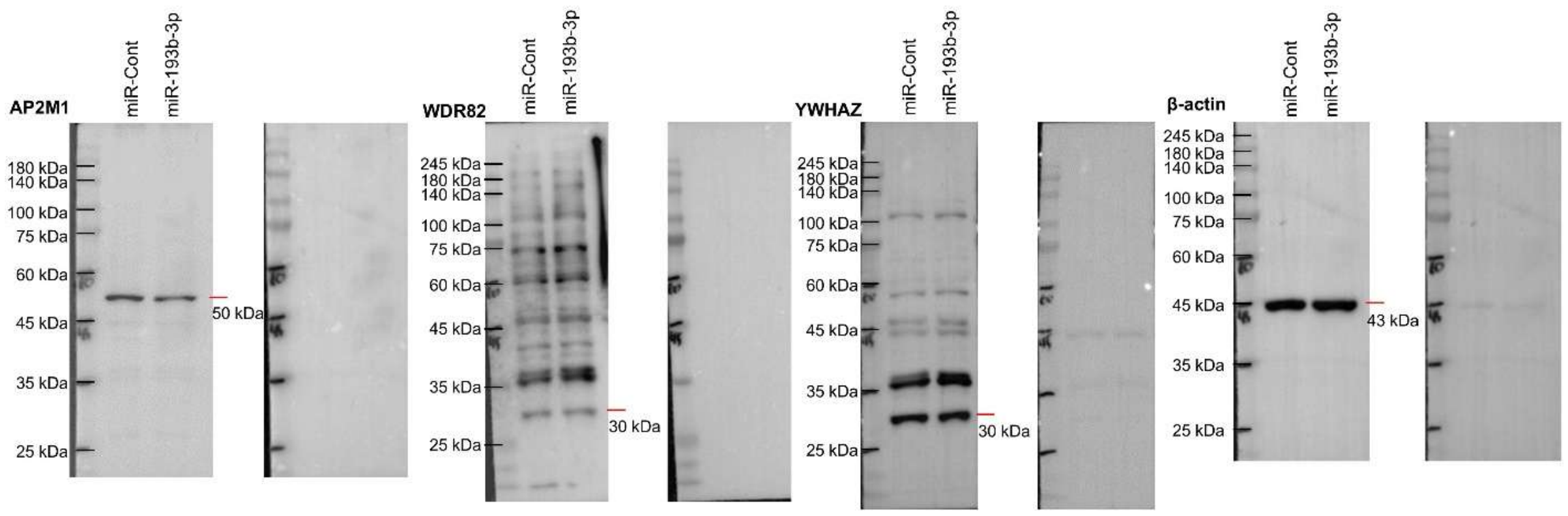
AP2M1
miR-Cont
miR-193b-3p
50 kDa
WDR82
30 kDa
YWHAZ
30 kDa
β-actin
43 kDa

D

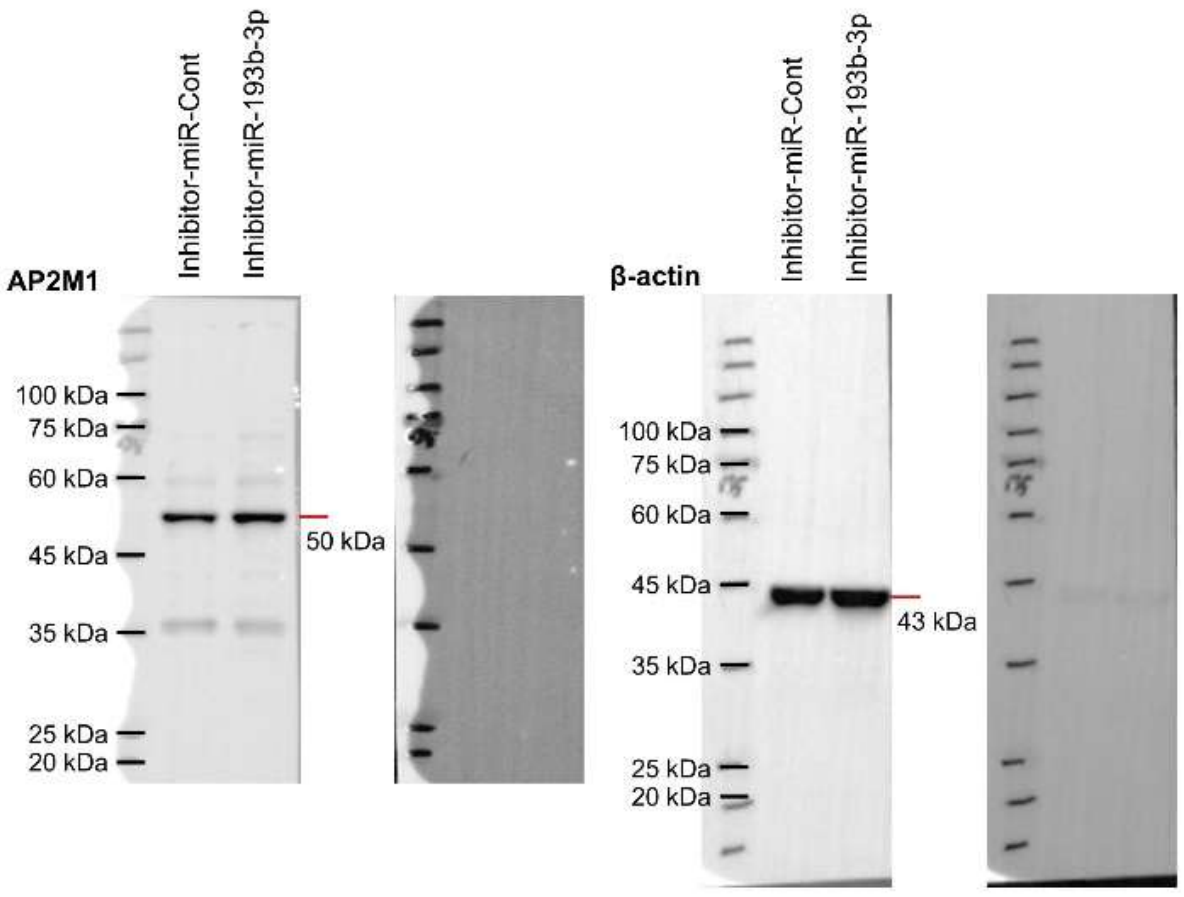

AP2M1
Inhibitor-miR-Cont
Inhibitor-miR-193b-3p
100 kDa
75 kDa
60 kDa
45 kDa
35 kDa
25 kDa
20 kDa
50 kDa
β-actin
Inhibitor-miR-Cont
Inhibitor-miR-193b-3p
100 kDa
75 kDa
60 kDa
45 kDa
35 kDa
25 kDa
20 kDa
43 kDa

E

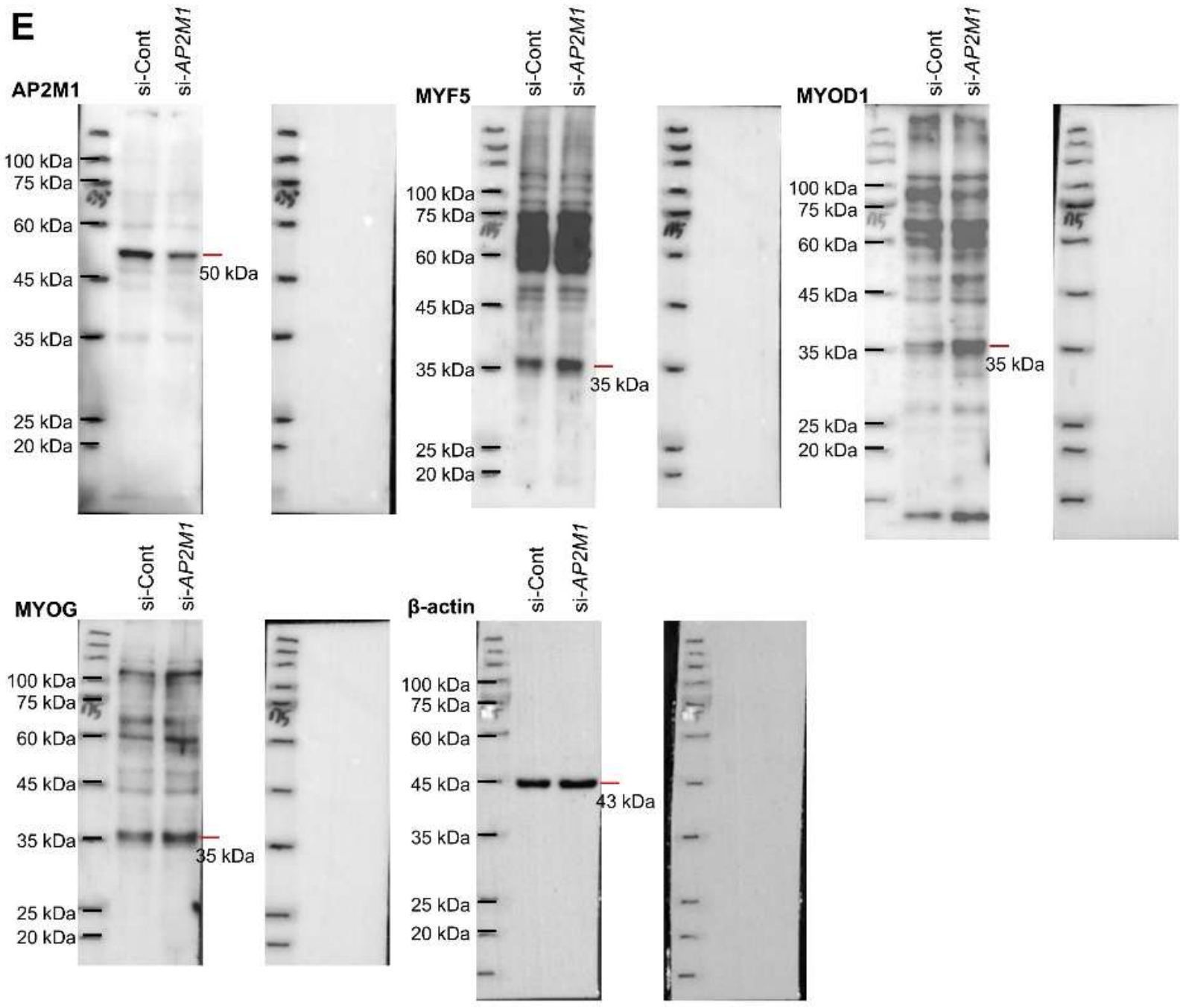

AP2M1
si-Cont
si-AP2M1
100 kDa
75 kDa
60 kDa
45 kDa
35 kDa
25 kDa
20 kDa
50 kDa
MYF5
si-Cont
si-AP2M1
35 kDa
MYOD1
si-Cont
si-AP2M1
35 kDa
MYOG
si-Cont
si-AP2M1
35 kDa
β-actin
si-Cont
si-AP2M1
43 kDa

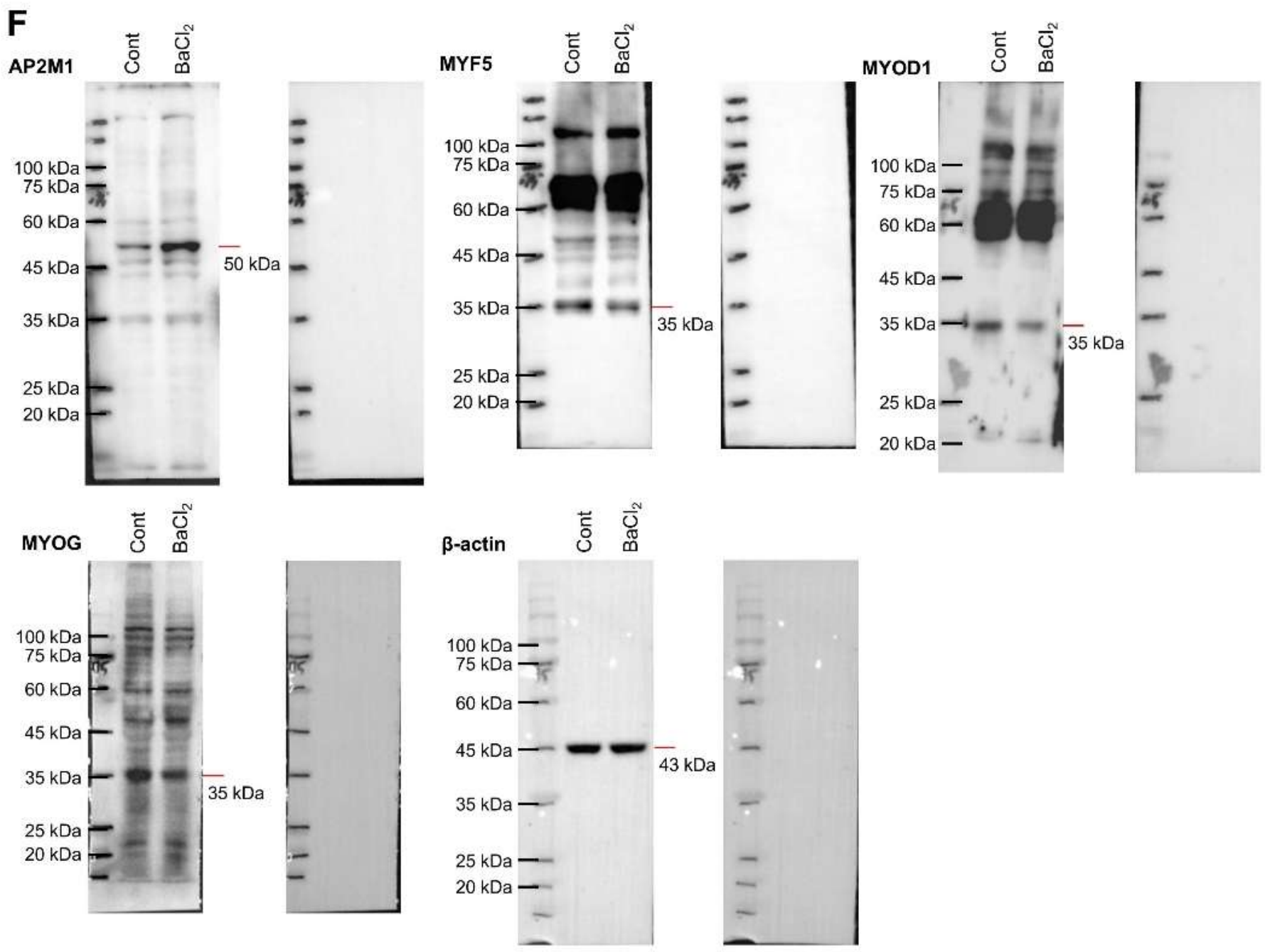

Figure S5. Uncropped western blot images correspond to the main figures.
